# Adaptations in electron transport chain complexes of clinical *Rhodococcus equi* revealed through comparative genomics

**DOI:** 10.1007/s11274-026-05110-w

**Published:** 2026-07-04

**Authors:** João Pedro Vasques da Conceição, Fabio Faria da Mota

**Affiliations:** 1 Computational and Systems Biology Laboratory, Oswaldo Cruz Institute, FIOCRUZ, Rio de Janeiro, Brazil; 2 Postgraduate Program in Computational Biology and Systems, Oswaldo Cruz Institute, FIOCRUZ, Rio de Janeiro, Brazil

**Keywords:** Anaerobic respiration, Comparative genomics, Pathogenesis, Respiratory chain, *Rhodococcus equi*, Virulence factors

## Abstract

*Rhodococcus equi* is an important veterinary and zoonotic pathogen that survives inside macrophage phagolysosomes. Nitrate reductase NarGHI is a known virulence factor for this pathogen. This enzyme complex serves as the terminal electron acceptor in anaerobic respiration in other pathogens, such as the phylogenetically related *Mycobacterium tuberculosis*. A recent study demonstrated that *R. equi* survives under anaerobic conditions. In this work, we endeavored to uncover the electron transport chain complexes of *R. equi*. By in silico comparison to known complexes in the Transporter Classification Database, 67 putative respiratory chain proteins were identified in *R. equi *103S. Their distribution was evaluated in the genomes of 37 clinical (animal and human infections) and 297 non-clinical (environmental) *Rhodococcus* isolates from the NCBI RefSeq database. NarGHI subunits were found in 100% of clinical and 35% of non-clinical genomes. Loci encoding four potential electron donor complexes (a membrane-bound formate dehydrogenase, a succinate dehydrogenase, and two Ni-Fe hydrogenases) were present in > 80% of clinical and < 50% of non-clinical isolates. Loci encoding a nitric oxide reductase, a potential terminal electron acceptor, were identified in 40.5% of clinical and 2% of non-clinical isolates. The results show that the respiratory chain in *R. equi* is highly complex, and point to potential metabolic adaptations of this species to hypoxic conditions.

**Supplementary Information:**

The online version contains supplementary material available at 10.1007/s11274-026-05110-w.

## Introduction

*Rhodococcus* bacteria are actinomycetes with diverse metabolic capabilities (Cappelletti et al. 2020). Recently, the *Prescottella* genus (Sangal et al. 2022) was reclassified as a subgenus within the genus *Rhodococcus* Zopf 1891, based on phylogenomic analyses (Val-Calvo et al. 2025). This subgenus harbors the mammalian pathogenic species *Rhodococcus equi* (formerly *Corynebacterium equi*, *Prescottella equi*, and *Rhodococcus hoagii*), which shows particular tropism for the respiratory system (Muscatello et al. 2007). Equids are the primary hosts for *R. equi*, which commonly causes respiratory tract infections. However, the pathogen demonstrates a broader host range, as it has also been documented to infect swine and ruminants (Witkowski et al. 2016; Żychska et al. 2021; Matsuoka et al. 2024). In humans, *R. equi* infections are mainly associated with immunocompromised individuals, such as those with HIV infection (Drancourt et al. 1992; Xu et al. 2022; Vandersnickt et al. 2024), although cases of infection in immunocompetent patients have also been reported (Lin et al. 2019). Like other *Rhodococcus* members, *R. equi* can survive and proliferate through multiple environmental conditions, with soil being its primary environmental reservoir, which contributes to its persistence and transmission potential (Von Bargen and Haas 2009).

Most studies on *R. equi* virulence focus on secreted proteins encoded by virulence plasmids. These proteins can arrest phagosomal maturation, allowing *R. equi* to survive within host macrophages’ phagosomes (Hondalus and Mosser 1994; Haubenthal et al. 2023). However, chromosome-encoded proteins have also been reported to contribute to *R. equi* virulence, including the siderophore rhequichelin involved in iron uptake (Miranda-CasoLuengo et al. 2012), mycoredoxins associated with oxidative stress resistance (Mourenza et al. 2019), and the respiratory nitrate reductase NarGHI, which catalyzes the reduction of nitrate (NO_3_^-^) into nitrite (NO_2_^-^) (Pei et al. 2007).

The NarGHI enzyme complex can be directly or indirectly involved in bacterial metabolism in multiple ways. Reduction of NO_3_^-^ is an essential step in the nitrate assimilation pathway, in which nitrogen is incorporated into bacterial biomass (González et al. 2006). NO_3_^-^ assimilation mediated by NarGHI has been observed in *Mycobacterium tuberculosis*, another intracellular respiratory pathogen in the Mycobacteriales order, where NO_2_^-^ is further reduced into ammonium (NH_4_^+^) by the nitrite reductase NirBD (Malm et al. 2009). *nirBD* genes were also predicted in the *R. equi* 103S genome (Letek et al. 2010). Disruption of the *narG* gene resulted in complete attenuation of *R. equi* infection in mice, indicating a major role in mammalian virulence (Pei et al. 2007). In contrast, disruption of *nirB* decreased *R. equi* survival in mice and macrophages but did not fully attenuate infection (Sangkanjanavanich et al. 2021). These findings indicate that *R. equi* NarGHI contributes to virulence by mechanisms beyond the assimilation of NO_3_^-^.

NarGHI is a membrane-bound enzyme complex that utilizes electrons from the quinone pool in the cytoplasmic membrane to reduce NO_3_^-^ and pump H^+^ across the membrane (Jormakka et al. 2003). Under hypoxia, this mechanism enables the use of NO_3_^-^ as the terminal electron acceptor in the respiratory chain, as observed in other bacterial pathogens, including *Pseudomonas aeruginosa* (Palmer et al. 2007), *Escherichia coli* (Kaviraj et al. 2024) and *M.* *tuberculosis* (Singh et al. 2020). In this context, NirBD primarily functions to prevent toxicity from the produced NO_2_^-^ (Page et al. 1990). During infection, pathogens may encounter hypoxic environments, such as inflamed tissues (Taylor and Colgan 2017) and macrophage phagolysosomes (James et al. 1998). Although *R. equi* is traditionally described as an aerobic organism (Prescott 1991; Sangal et al. 2022), microaerophilic and anaerobic growth on sheep blood agar has recently been observed (Dos Santos et al. 2025). This may indicate that the role of NarGHI in *R. equi* virulence is also related to anaerobic respiration under the hypoxic conditions encountered during infection.

During respiration, the electrons used for terminal acceptor reduction are provided to the quinone pool through oxidation of various donor molecules. This oxidation is mediated by other membrane-bound enzyme complexes, forming redox loops (Kaila and Wikström 2021). Bacterial aerobic respiratory chains are typically powered by electrons released by the oxidation of NADH via NADH dehydrogenase (Complex I) (Kaila and Wikström 2021) and succinate via succinate dehydrogenase (Complex II) (Iverson et al. 2023). Bacteria can also use alternative electron donors, including formate, hydrogen, and methanol (Kaila and Wikström 2021). Anaerobic nitrate respiration via NarGHI is most commonly coupled to formate oxidation (Jormakka et al. 2003). If NarGHI constitutes an important component of the electron transport chain in *R. equi*, other complexes participating in this redox loop must also be elucidated.

The Transporter Classification Database (TCDB) is a curated resource that compiles representative proteins for the Transporter Classification (TC) system, which classifies membrane transport proteins (Saier et al. 2021). This system includes transmembrane electron carriers (TC 5), such as NarGHI, and oxidoreduction-driven transporters (TC 3.D), such as NADH-quinone oxidoreductases (Saier et al. 2016). In the present study, we searched for respiratory chain complexes in the *R. equi* 103S genome by comparison with reference proteins from TCDB, followed by comparative genomic analysis to examine their distribution across *Rhodococcus* with known isolation sources. Through this in silico analysis, five putative electron transport chain complexes were identified, including a membrane-bound formate dehydrogenase (FDH), a succinate dehydrogenase, two Ni-Fe hydrogenases complexes, and a nitric oxide reductase.

## Materials and methods

### Genomic data and metadata retrieval

To analyze the distribution of respiratory chain subunits, we leveraged publicly available genome data to compare clinical and non-clinical isolates. The genomic data and metadata of *Rhodococcus* isolates available on the NCBI RefSeq database (O’Leary et al. 2016) were queried on 23 May 2023. In total, 650 *Rhodococcus* genomes were available. These genomes were categorized as clinical or non-clinical isolates based on the isolation source metadata available in the NCBI Biosample database (Barrett et al. 2012). Genomes for which the isolation source was not available or inconclusive were excluded from the analysis. In total, 334 *Rhodococcus* genome assemblies were selected, including 37 clinical and 297 from non-clinical isolates, and their genomic data were queried (Table S1).

### Identification of enzyme complexes involved in the electron transport chain through TCDB homology analysis

Sequences for 678 reference proteins from the TC 3.D subclass and 265 reference proteins from the TC 5 class were found in the TCDB database (https://www.tcdb.org) (Saier et al. 2016) and are shown in Table S2. These reference proteins served as queries for comparative analysis against predicted protein sequences from the *R. equi* 103 S genome. Sequence comparisons were performed using BLASTp v2.13.0 (Camacho et al. 2009), with homology relationships determined using reciprocal best-hit methodology (Hernández-Salmerón and Moreno-Hagelsieb 2020). The results were filtered to exclude those with an e-value > 1e-5, a well-established threshold for identifying homologs (Gibbons et al. 2015). This threshold is commonly employed when searching for homologs in the TCDB database (Anwar et al. 2025; Rao et al. 2026). Visualizations for BLAST alignment results were created using the web tool Kablammo (Wintersinger and Wasmuth 2015). TCDB contains only one reference sequence per TC classification. Therefore, for some proteins, a search for closer homologs in the non-redundant protein sequences database (nr) was also conducted using the BLAST web service (Camacho et al. 2009). Conserved domains were identified using the predicted amino acid sequences and the online tools NCBI CD-Search (Wang et al. 2023) and InterProScan (Jones et al. 2014).

### Gene orthology and phylogenetic tree inference

Orthology analysis was used to evaluate the phylogenetic distribution of enzyme complex homologs across *Rhodococcus* genomes. Orthologous protein groups (OGs) were inferred from predicted protein sequences of the 334 genomes from RefSeq using OrthoFinder 2.5.4 with default parameters, including Diamond and DendroBLAST methods (Emms and Kelly 2019). OrthoFinder was also used to infer the rooted phylogenetic species tree (Emms and Kelly 2017, 2018). Data from OGs was also used to evaluate the co-occurrence of identified respiratory complex homologs. Co-occurrence was quantified utilizing pairwise Pearson correlation coefficients, which were calculated using the “corr” function from the Pandas Python package. Visualization of these correlations was created using the Seaborn package in Python (Waskom 2021).

### Genomic context and synteny analysis

Genomic context analysis of genes encoding putative enzyme complex homologs was conducted through synteny comparisons between genomes from *Cupriavidus necator* H16, *Mycobacterium smegmatis* MC2 155, *Mycobacterium tuberculosis* H37Rv, *Pyrococcus furiosus* DSM 3638, the reference clinical isolate *R. equi* 103 S, and *Rhodococcus rhodochrous* EP4 (Table S3). Synteny analysis was performed using the Python package pyGenomeViz v1.6.1 with the reciprocal best-hit methodology and protein identities were calculated using MMseqs2 (Mirdita et al. 2021).

### Analysis of predicted membrane-bound formate dehydrogenase

Different analyses were performed to determine whether the putative FDH heterotrimer enzyme found in *Rhodococcus* genomes is bound to the cytoplasmic membrane. For each putative subunit, protein topology and hydrophobic regions predictions were conducted using Deep-TMHMM (Hallgren et al. 2022). Subcellular location prediction was performed using PSORTb v3.0.3 (Yu et al. 2010).

To classify the putative FDH enzyme of *R. equi* 103 S within the classification system proposed by Nielsen et al. (2019), its amino acid sequence was compared against 23 biochemically characterized bacterial FDHs spanning five of the six defined types: 2 (Type 1), 11 (Type 2), 4 (Type 4), 5 (Type 5), and 1 (Type 6). Type 3 was excluded from the analysis, as no representative sequences were identified by Nielsen et al. (2019). Multiple sequence alignment using the catalytic FDH-α subunits from these 23 previously characterized FDHs and the putative FDH-α subunit from *R. equi* 103S (WP_162096942.1) was performed using ClustalW v2.1 (Larkin et al. 2007). The sequences used for phylogenetic analysis were not trimmed. Multiple sequence alignment visualization was created using JalView v2.11.4.1 (Waterhouse et al. 2009). The resulting alignment was used as input to infer a maximum-likelihood phylogenetic tree with IQ-TREE v3.0.1 with 1,000 bootstrap replicates (Trifinopoulos et al. 2016). Tree visualization was created using the web-based iTOL v7 platform (Letunic and Bork 2024). Additionally, all 24 FDH-α subunits were subjected to signal peptide and transmembrane topology prediction using SignalP 6.0 (Teufel et al. 2022) and Deep-TMHMM (Hallgren et al. 2022), respectively.

For further characterization, the three-dimensional (3D) structure for the enzyme was predicted with AlphaFold3 (Abramson et al. 2024). The structure was predicted for the *R. equi* 103S putative FDH heterotrimer with proteins FDH-α (WP_162096942.1, 1080 amino acids), FDH-β (WP_013415246.1, 315 amino acids), and the NrfD family protein (WP_041673985.1, 330 amino acids). To create a model that accurately represents the mature FDH heterotrimer, the first 28 amino acids of the FDH-α sequence, corresponding to the cleavage of the putative twin-arginine signal peptide, were removed. To check if the predicted structure possesses the characteristics found in other FDH heterotrimers, the *R. equi* putative FDH heterotrimer structure was compared to the experimentally resolved structures of the *Escherichia coli* FDH-N heterotrimer (PDB 1KQF) (Jormakka et al. 2002) and the *Megalodesulfovibrio gigas* FDH dimer (PDB 1H0H) (Raaijmakers et al. 2002) using the “cealing” command in PyMol v3.1. Amino acid interactions predicted by AlphaFold3 served as the basis for predicting regions of interface between proteins using AlphaBridge, with an interaction cutoff of 0.9 in the Predicted Distance Error (PDE) metric (Álvarez-Salmoral et al. 2024).

## Results

### *R. equi* proteins homologous to respiratory complexes from TCDB

After comparisons between reference respiratory complexes from TCDB and *R. equi* 103S predicted proteins, 53 homologous proteins were found in the TC 3.D subclass and 14 homologous proteins in the TC 5 class (Table S4). These proteins include subunits for three canonical respiratory complexes in aerobic organisms (Marreiros et al. 2016): 13 NADH-quinone oxidoreductase subunits (complex I), 6 cytochrome c oxidase subunits (complex III), and the aerobic terminal cytochrome *bd* oxidase (complex IV) (Fig. S1). By inferring orthology among predicted proteins from 334 *Rhodococcus* genomes, these subunits were found widespread across analyzed genomes (Table S4). Non-canonical respiratory complexes were also found to be widespread in the *Rhodococcus* genus. Three putative transhydrogenase subunits (WP_037084388.1, WP_005514102.1 and WP_013415590.1) were identified as homologs of the proton-translocating transhydrogenase complex (P0C186, P0C187 and P0C188) found in *Rhodospirillum rubrum.* Two putative electron transfer flavoprotein subunits (WP_013416629.1 and WP_005518976.1) were identified as homologs of the FixA (Q6US87) and FixB (Q6US86) subunits from the nitrogen fixation complex FixABCX, also found in *R. rubrum* (Table S4, Fig. S2).

As expected, comparisons show that the NarGHI complex in *R. equi* 103 S is homologous to membrane-bound nitrate reductase complexes NarGHI and NarZYV found in *E. coli* (Table 1). This enzyme was found in all clinical-origin genomes, but in only 35% of non-clinical genomes (Table S4). The non-clinical genomes harboring the NarGHI complex are from the species *Rhodococcus gordoniae*,* Rhodococcus pyridinivorans*, *Rhodococcus rhodochrous*, and *Rhodococcus ruber* (Fig. S3). Excluding the NarGHI subunits, only 15 other proteins were found in < 50% of non-clinical genomes (Table 1). Within these 15 proteins, a putative GlxC (WP_013417126.1) and an FAD-dependent oxidoreductase (WP_013416252.1) presented very low coverage in the alignment, 28% and 12%, respectively (Table 1). Based on comparisons of the conserved domains with their TCDB homologs, these proteins were deemed to be unrelated to the respiratory chain and were excluded from further analysis (Figs. S4 and S5) (Table 2).

**Table 1:** *R. equi* 103S proteins homologous to respiratory complex proteins deposited in TCDB and found in less than 50% of non-clinical genomes

Putative protein complex	*R*. equi 103S protein accession	*R*. equi 103 S RefSeq protein annotation	*R*. equi 103S number of amino acids	TCDB homolog accession	Annotation for TCDB homolog	Number of amino acids for TCDB homolog	Occurrence in 334 *Rhodococcus* genomes		E-value	Identity (%)	Coverage (%)
							Clinical (total 37) (%)	Non-clinical (total 297) (%)			
Nitrate reductase NarGHI	WP_013414677.1	nitrate reductase subunit alpha	1238	P09152	NarG	1247	100	35	0	48	97
	WP_013414676.1	nitrate reductase subunit beta	560	P19318	NarY	514	100	35	0	54.5	87
	WP_013414674.1	respiratory nitrate reductase subunit gamma	255	P0AF32	NarV	226	100	35.7	5.83e-32	31.5	88
Formate dehydrogenase	WP_162096942.1	formate dehydrogenase	1080	P24183	FdnG	1015	97.3	9.8	0	38.8	88
	WP_013415246.1	4Fe-4 S dicluster domain-containing protein	315	P0AAJ3	FdnH	294	83.8	20.5	1.49e-48	35.7	93
	WP_041673985.1	NrfD family protein	330	P31077	Polysulfide reductase chain C	317	100	10.1	8.17e-06	27.2	81
Ni-Fe hydrogenase 3b	WP_005514364.1	nickel-dependent hydrogenase large subunit	439	P60227	F420-non-reducing hydrogenase subunit A	472	91.9	18.9	3.44e-40	27.6	96
	WP_013415447.1	oxidoreductase	263	P60239	F420-non-reducing hydrogenase subunit G	308	91.9	19.5	2.53e-25	27.6	78
Ni-Fe hydrogenase 1h	WP_005517441.1	nickel-dependent hydrogenase large subunit	597	P31883	Quinone-reactive Ni/Fe-hydrogenase large chain	576	91.9	42.1	4.63e-44	26.1	94
	WP_013417287.1	hydrogenase expression protein HypE	361	Q50248	VHOG	383	91.9	43.8	2.58e-31	30.2	94
	WP_013417278.1	hydrogenase formation protein HypD	376	OLS30727	Hydrogenase formation protein HypD	359	91.9	43.8	4.03e-102	45.6	83
	WP_005517438.1	hydrogenase maturation nickel metallochaperone HypA	109	P0A703	Hydrogenase maturation factor HybF	113	91.9	45.1	6.31e-14	34.8	99
	WP_005517454.1	HypC/HybG/HupF family hydrogenase formation chaperone	90	P0AAM7	Hydrogenase maturation factor HybG	82	91.9	44.8	1.18e-18	44.9	84
	WP_013417281.1	hydrogenase maturation protease	165	P37182	Hydrogenase 2 maturation protease	164	91.9	44.1	1.34e-09	32.4	92
Sdh1	WP_013416316.1	succinate dehydrogenase/fumarate reductase iron-sulfur subunit	251	Q65GF5	SdhB	254	100	36.4	8.65e-24	29.3	85
NO reductase	WP_013414602.1	nitric-oxide reductase large subunit	777	K6C808	Nitric-oxide reductase qNorB	787	40.5	2	8.28e-158	37.6	97
-	WP_013417126.1	protein glxC	232	O27600	FwdC	270	91.9	33	5.87e-08	43.1	28^a^
-	WP_013416252.1	FAD-dependent oxidoreductase	506	Q9ZR03	Cytochrome b6-f complex iron-sulfur subunit	229	100	42.1	7.18e-11	44.1	12^a^

(^a^) Partial coverage only

**Table 2:** Summary of possible roles for each putative electron transport complex found conserved in *Rhodococcus* genomes

Putative protein complex	Role	References
NarGHI	Use NO_3_^+^ as an electron acceptor, taking electrons from the quinone pool. Reduce NO_3_^+^, produce NO_2_^+^.	Jormakka et al. (2003);Coelho and Romão (2015)
NirBD	Reduces NO_2_^+^, produces NH_4_^+^. Removes toxic NO_2_^+^. NH_4_^+^ may be assimilated as a nitrogen source, through production of glutamate. NH_4_^+^ may also be secreted from the cell, increasing the environment’s pH.	Page et al. (1990); Bueno et al. (2018)
NO reductase	Use NO as an electron acceptor, taking electrons from quinone pool. Reduces NO, produces N_2_O. Reduction of NO may avoid production of RNS in host cells.	Gopalasingam et al. (2019)
Formate Dehydrogenase	Oxidizes formate and transfers electrons to quinone pool. Main electron donor associated with NarGHI in *E. coli*.	Unden et al. (2014)
Succinate dehydrogenase 1	Related to aerobic respiration in *Mycobacterium*. No fumarate reductase activity in *Mycobacterium*. Oxidizes succinate, produces fumarate.	Hartman et al. (2014); Pecsi et al. (2014)
Succinate dehydrogenase 2	Related to anaerobic respiration in *Mycobacterium*. No fumarate reductase activity in *Mycobacterium*. Oxidizes succinate, produces fumarate.	Hartman et al. (2014); Pecsi et al. (2014)
Ni-Fe hydrogenase 1h	Oxidizes H_2_. Possibly transfers electrons to quinone pool.	Greening et al. (2015)
Ni-Fe hydrogenase 3b	Reduces 2 H^+^, producing H_2_. In *M. smegmatis* transcription and function are related to hypoxia. Possibly coupled to Ni-Fe 1 h. Production of H_2_ may also help raise pH.	Berney et al. 2014a); Lin and Liaw (2020)

The remaining 13 proteins had higher alignment coverage with their corresponding TCDB homologs, and their predicted functions were more closely related to those of their homologs (Table 1). This group includes the large subunit of a nitric oxide reductase (WP_013414602.1) that is primarily found in the pathogenic *R. equi* branch, along with two non-clinical genomes, identified as *Rhodococcus defluvii* and *Rhodococcus* sp. (Fig. S3, highlighted in pink). In addition to the pathogenic *R. equi* branch, the other 12 proteins were also found in certain phylogenetic clades of non-clinical isolates (Fig. S3, highlighted in other colors; Table S5).

Pearson correlation analysis revealed a high degree of co-occurrence among genes encoding subunits of the same enzyme complexes, such as NarGHI (Fig. S6; Table S6). Two genes homologous to FDH subunits and an NrfD family protein exhibited high co-occurrence values among themselves and with the nitrate reductase NarGHI complex (Fig. S6, highlighted as a dark blue square over the heatmap). Genes related to Ni-Fe hydrogenases formed two different clusters. Genes across these clusters presented higher co-occurrence values with each other than when compared to genes in other clusters (Fig. S6, highlighted as a green square over the heatmap).

### Membrane-bound formate dehydrogenase

**Fig. 1:**
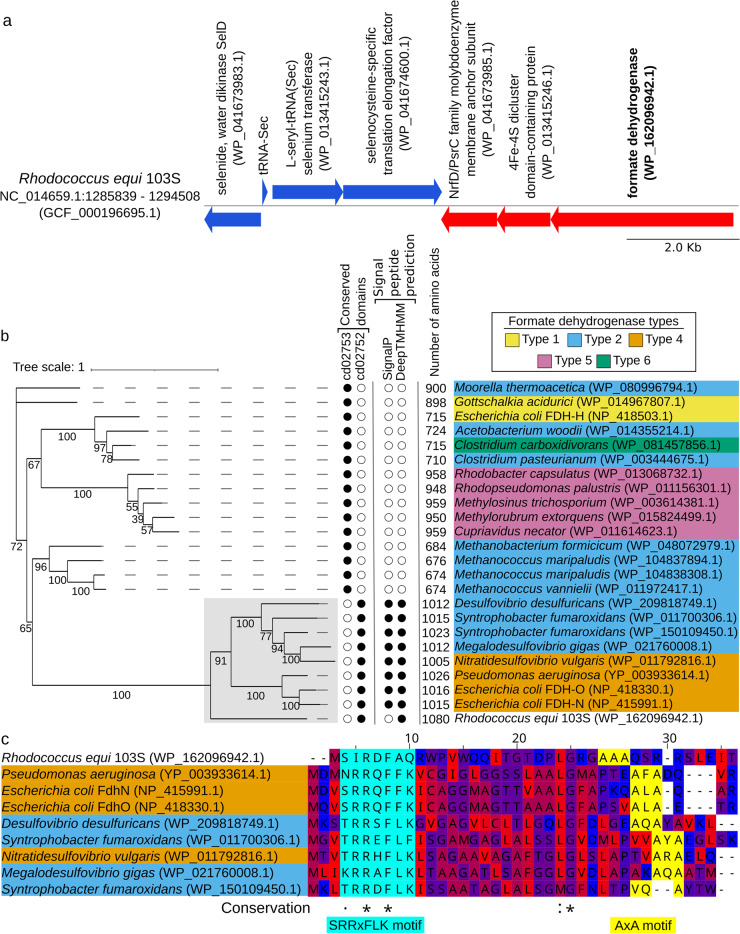
Genomic and phylogenetic analysis of *R. equi* 103S putative FDH. **a**: Putative FDH gene cluster in *R. equi* 103S genome. Blue arrows: seleno-cysteine biosynthesis genes; Red arrows: putative FDH genes. Gene functional annotations retrieved from the RefSeq database. **b**: Maximum likelihood phylogenetic tree inferred with 23 reference FDH-α. Bootstrap values are found on each node. Black circles at tree leaves represent predictions of cd02753 and cd02752 conserved domains, as well as the presence of a signal peptide by SignalP and DeepTMHMM. Clade containing *R. equi* FDH-α is highlighted in grey. Label background colors represent the types of FDH as established in the literature (Nielsen et al. 2019). **c**: Multiple sequence alignment of FDH-α sequences in the same clade as *R. equi* FDH-α. Amino acids are colored according to hydrophobicity. Cyan and yellow highlighting indicate residues of the predicted SRRxFLK and AxA motifs, which are the sites for recognition and cleavage by the Tat translocase, respectively. Label background colors denote FDH types. Blue: Type 2; Orange: Type 4

Through the comparison with proteins deposited in TCDB, a putative FDH catalytic (FDH-α) subunit (WP_162096942.1) and a 4Fe-4 S dicluster-containing protein (FDH-β) (WP_013415246.1) from *R. equi* 103 S were determined to be homologs to the FDH-α subunit (TCDB accession P24183) and FDH-β subunit (TCDB accession P0AAJ3) of membrane-bound periplasmic FDHs found in *Escherichia coli* (Table 1). The genes encoding these proteins are found in the same gene cluster in the *R. equi* 103 S genome (NC_014659.1:1285839–1295219). Alongside them, the gene encoding the seleno-cysteine tRNA (REQ_RS06040) and three genes encoding proteins related to seleno-cysteine biosynthesis (WP_041673983.1, WP_013415243.1 and WP_041674600.1) were also found (Fig. 1A). As it is not one of the 20 standard amino acids, a search for the seleno-cysteine codon, TGA, was performed in all the predicted coding sequences of the *R. equi* 103 S genome. An in-frame TGA codon was found only in the coding sequence for the putative FDH-α subunit (WP_162096942.1).

*E. coli* membrane-bound FDHs also have a third subunit, FDH-γ, which anchors the enzyme to the membrane and transfers electrons to the quinone pool (Jormakka et al. 2002). No homologs of the *E. coli* FDH-γ subunit were found in the *R. equi* 103S genome. However, in the *R. equi* 103S gene cluster, alongside putative FDH-α and FDH-β subunits, there is also a third gene predicted to encode a protein from the NrfD protein family (Fig. 1A). Proteins in this family serve as membrane anchors for various energy-transducing enzymes (Calisto and Pereira 2021). Comparison with TCDB identified this putative NrfD family protein as a homolog of the polysulfide reductase chain C found in *Wolinella succinogenes* (Table 1). Given that its encoding gene is located adjacent to the putative FDH-α and FDH-β subunits, we hypothesize that the putative NrfD family protein may actually play a role similar to FDH-γ, binding *R. equi* 103S FDH to the membrane and transferring electrons to the quinone pool.

Through analysis with PSORTB, both the putative FDH-β subunit and NrfD family protein from *R. equi* 103S were predicted to be bound to the cytoplasmic membrane, while the putative FDH-α subunit was predicted to be a cytoplasmic protein (Table S7). DeepTMHMM analysis classified the FDH-α subunit as a globular protein containing a signal peptide in the N-terminal, suggesting it may actually be exported to the extracellular space (Fig. S7A; Table S7). Analysis with SignalP did not predict any signal peptide (Fig. S7B). NrfD family protein and FDH-β subunit were identified as α-helical transmembrane proteins lacking signal peptides, but containing extracellular and cytoplasmic regions. While FDH-β contains 1 predicted transmembrane region, NrfD contains 8 (Fig. S7A; Table S7).

Nielsen et al. (2019) previously proposed a classification system grouping FDH enzymes into six types based on structural and biochemical characteristics, including the presence or absence of the membrane-anchoring FDH-γ subunit. To determine where the *R. equi* 103S FDH enzyme fits within this framework, we inferred the phylogenetic relationships between its putative FDH-α subunit and those of the 23 experimentally characterized FDHs classified by Nielsen et al. (2019). In the resulting phylogenetic tree, the clustering of FDH proteins was inconsistent with their Type classification, with the notable exception of Type 5 FDHs (Fig. 1B). Type 2 FDHs displayed the broadest phylogenetic distribution. Type 2 FDHs from archaea *Methanobacterium formicicum*, *Methanococcus maripaludis*, and *Methanococcus vannielii* were grouped separately from bacterial Type 2 FDHs (Fig. 1B). The *R. equi* 103S FDH-α subunit was positioned within a clade comprising all Type 4 FDHs and four Type 2 FDHs found in dissimilatory sulfate-reducing bacteria (Fig. 1B, clade highlighted in grey). Conserved domain prediction by CD-Search indicates that the nine FDH-α subunits in this clade possess the formate dehydrogenase conserved domain cd02752, while the other 15 FDH-α subunits possess the formate dehydrogenase conserved domain cd02753. (Fig. 1B).

Among the FDHs clustering with *R. equi* 103S is the FDH-α subunit of *E. coli* FDH-N (Fig. 1B), which is known to carry a twin-arginine translocation (Tat) signal peptide that directs its export to the periplasm (Bogsch et al. 1998; Sargent et al. 1999). To further understand the relationship between signal peptides and FDHs, SignalP 6.0 and Deep-TMHMM predictions were performed for all FDH-α subunits included in the phylogenetic analysis. Both tools predicted the presence of a signal peptide in all eight FDH-α subunits in the same clade as *R. equi* FDH-α (Fig. 1B). SignalP specifically predicted that these signal peptides contain the Tat motif. A closer examination of the multiple sequence alignment within this clade revealed that the *R. equi* 103 S FDH-α subunit contains two arginine residues that may correspond to the AxA cleavage motif (Fig. 1C), found in the polar region of Tat signal peptides (Palmer and Stansfeld 2020). It also possesses residues closely resembling the recognition motif SRRxFLK of Tat signal peptides, including conserved serine, arginine, and phenylalanine residues (Fig. 1C). Across the aligned sequences, the serine residue is the least conserved, while the second arginine residue is the most conserved (Fig. 1C). Notably, the region between the SRRxFLK and AxA motifs in the *R. equi* 103 S FDH-α subunit is shorter and possesses a more polar composition relative to the other FDH-α in the clade, while still retaining a conserved glycine residue (Fig. 1C).

Membrane translocation and cleavage of Tat signal peptides are mediated by the twin-arginine translocase, which contains three subunits, TatA, TatB, and TatC (Palmer and Stansfeld 2020). Proteins containing the domains corresponding to TatA, TatB, and TatC subunits were identified in the *R. equi* 103S genome through CD-Search (Table S8). In *Nitratidesulfovibrio vulgaris*, dimeric FDH complexes exported to the periplasm are also able to transfer electrons to the quinone pool through interaction with the QrcABCD complex (Venceslau et al. 2010). QrcABCD complex homologs have previously been identified in *Megalodesulfovibrio gigas* (Morais-Silva et al. 2014). No homologs for the *N. vulgaris* QrcABCD complex (WP_010940677.1, WP_271370570.1, WP_010937996.1 and WP_010937995.1) were found in the 334 *Rhodococcus* genomes analyzed.

**Fig. 2:**
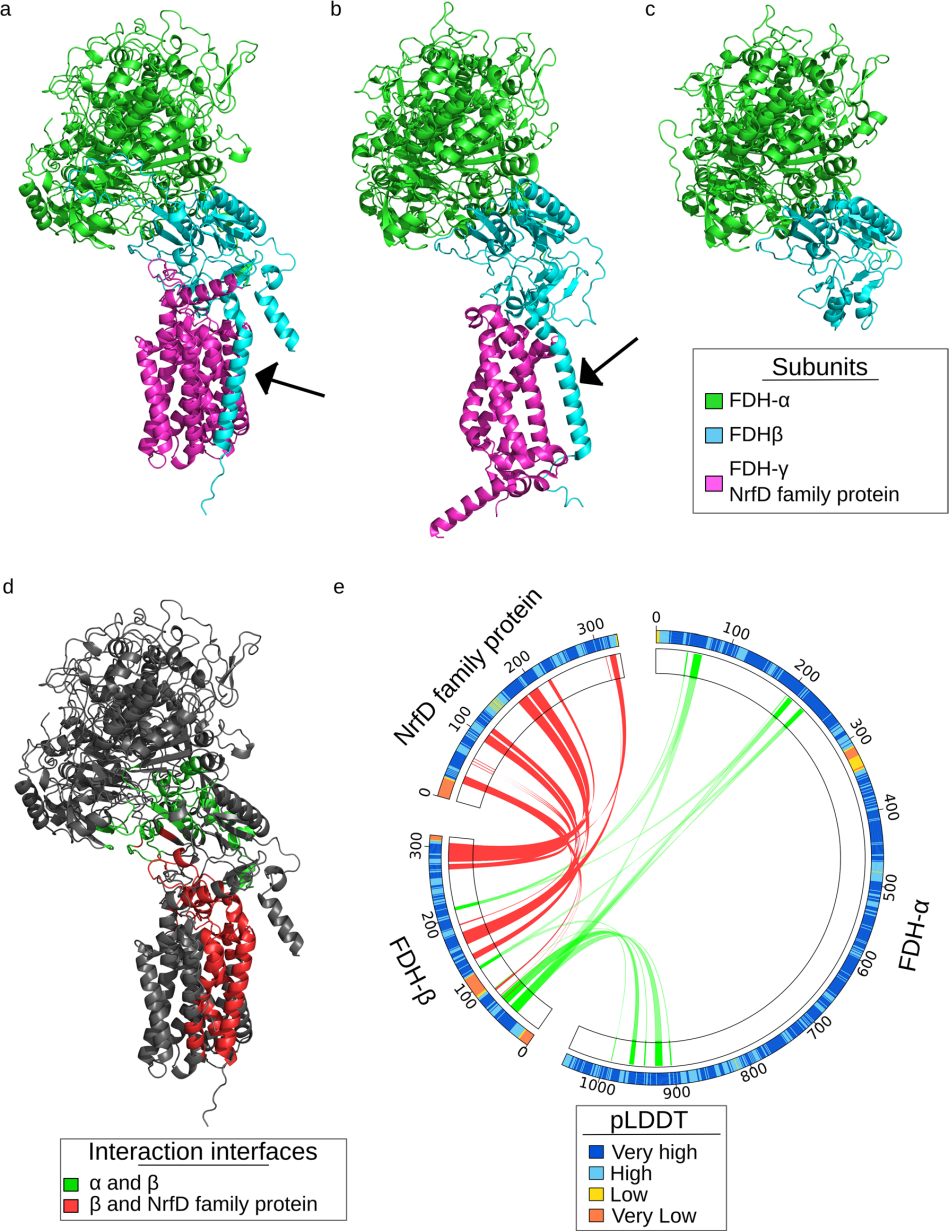
Analysis of *R. equi* 103 S FDH predicted structure. Green: FDH-α; Cyan: FDH-β; Magenta: FDH-γ or NrfD family protein. **a**: *R. equi* 103S FDH structure as predicted by AlphaFold3. **b**: Experimentally solved *E. coli* FDH (PDB 1KQF). Black arrows point to the FDH-β C-terminal alpha-helix. **c**: Experimentally solved *Megadesulfovibrio gigas* FDH (PDB 1H0H). **d**: Regions of interaction interfaces between *R. equi* 103 S FDH subunits, as predicted by AlphaBridge. Colored regions indicate the interaction interfaces. Green: FDH-α and FDH-β interface; Red: FDH-β and NrfD family protein interface. **e**: Circos plot demonstrating predicted interactions between residues in different *R. equi* 103 S FDH subunits. Chord diagram showcases predicted interactions between residues. Their colors correspond to the interface regions predicted by AlphaBridge. The outer circle showcases pLDDT values for each residue in the predicted structure. Blue: Very high confidence (plDDT > 90); Cyan: High confidence (90 > plDDT > 70); Yellow: Low confidence (70 > plDDT > 50); Orange: Very low confidence (50 > plDDT)

Given that phylogenetic inference could not definitively classify the type of FDH in *R. equi* 103S, the 3D structure of the enzyme complex was predicted and analyzed. The enzyme was modeled as a heterotrimer of putative FDH-α, FDH-β, and the NrfD family protein (Fig. 2A). For this predicted structure, the pTM score was 0.89, and the ipTM score was 0.88. Individual amino acids presented high pLDDT scores for most of the structure (Fig. S8A and S8B). This 3D structural model predicted interactions between residues of subunits FDH-α and FDH-β and residues of the FDH-β subunit and the NrfD family protein (Figs. 2D and 2E).

**Fig. 3:**
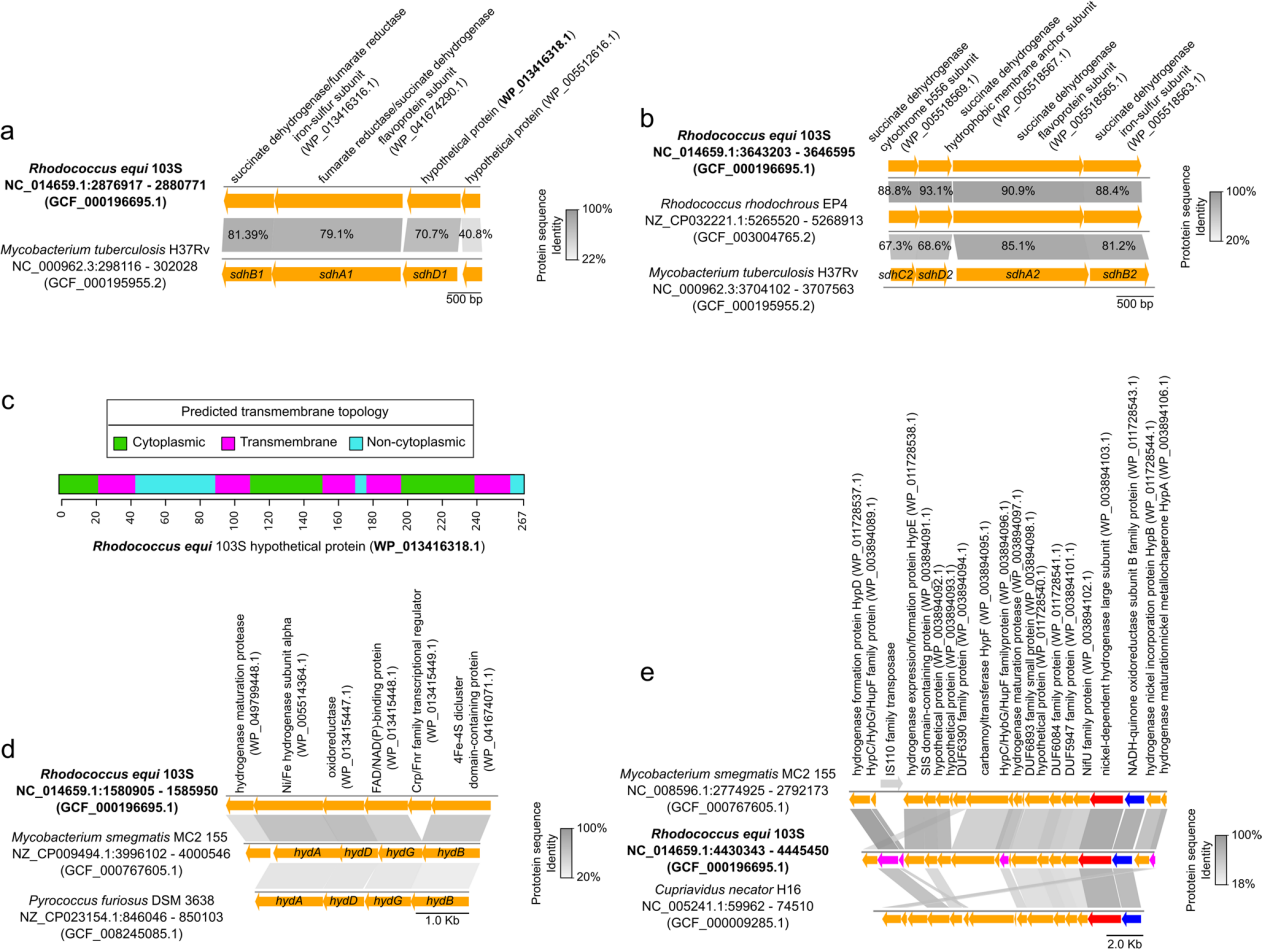
Genomic and synteny analysis for genes encoding the predicted succinate dehydrogenases and Ni-Fe hydrogenase in *R. equi* 103S. **a**: Synteny between genes encoding SDH1 in *R. equi* 103S and *M. tuberculosis* and, **b**: Synteny between genes encoding SDH2 in *R. equi* 103S, the non-clinical genome *R. rhodochrous* EP4, and *M. tuberculosis*. **c**: Transmembrane topology predicted with PHOBIUS for the possible intermembrane subunit (WP_013416318.1) of putative SDH1D from *R. equi* 103S. Green regions: Cytoplasmic; Magenta: Transmembrane; Cyan: Non-cytoplasmic. **d**: Synteny between genes encoding Ni-Fe hydrogenase 3b in *R. equi* 103S, *M. smegmatis* and *Pyrococcus furiosus*. **e**: Synteny between genes encoding Ni-Fe hydrogenase 1h in *R. equi* 103S, *M. smegmatis* and *Cupriavidus necator*. Red arrows: large subunit *hhyL*; Blue arrows: small subunit *hhyS*; Magenta arrows: homologs to Ni-Fe hydrogenase maturation proteins deposited in TCDB. Each arrow represents a gene. Grey trapeziums drawn between sequences indicate the reciprocal best hits relationship between encoded proteins. The grey scale of the trapeziums represents the sequence identity between protein amino acid sequences. Gene functional annotations retrieved from the RefSeq database

The 3D structural model of *R. equi* 103S putative FDH predicted by AlphaFold3 was compared to the experimentally solved structures of the heterotrimeric Type 4 FDH-N from *E. coli*, constituted of FDH-α, FDH-β, and FDH-γ subunits (Fig. 2B), and the heterodimeric Type 2 FDH from *M. gigas*, constituted of FDH-α and FDH-β subunits (Fig. 2C). In the phylogenetic inference, the FDH-α subunits from *E. coli* and *M. gigas* were both clustered within the same phylogenetic clade as the *R. equi* 103S putative FDH-α subunit (Fig. 1B, clade highlighted in grey). In the structural alignments involving *E. coli* and *M. gigas* FDH complexes, the RMSD values were 1.89 Å and 2.64 Å, respectively (Fig. S8C and S8D).

As predicted by DeepTMHMM, residues in the C-terminal region of the *R. equi* FDH-β subunit showed an α-helix conformation (Fig. 2A, black arrow). This α-helix is also present in the C-terminus of the *E. coli* FDH-β subunit (Fig. 2B, black arrow), but is absent in the *M. gigas* FDH-β subunit (Fig. 2C). Predicted residue interactions indicate that the α-helix is an important part of the interface between the *R. equi* 103S putative FDH-β subunit and NrfD family protein (Fig. 2D and E). The structure of the NrfD family protein as predicted by AlphaFold3 contains 8 α-helices, which was also predicted by DeepTMHMM (Fig. 2A), while the *E. coli* FDH-γ subunit contains only 4 α-helices (Fig. 2B). FDH complex from *M. gigas* does not have the membrane anchor FDH-γ subunit (Fig. 2C).

### Two distinct succinate dehydrogenase enzyme complexes

The search for homologs in TCDB found two distinct succinate dehydrogenase (SDH) systems in *R. equi* 103S. The first system consists of four subunits homologous to SDH2 found in *M. smegmatis*, and is present in both clinical and non-clinical *Rhodococcus* genomes (Table S4). The genes encoding these subunits were found in a gene cluster in the *R. equi* 103S genome (NC_014659.1:3643203–3646595). This gene cluster is also found in the genome of the non-clinical *Rhodococcus rhodochrous* strain EP4 (NZ_CP032221.1:5265520–5268913), the type species of the *Rhodococcus* genus (Fig. 3B).

In the second system, only the iron-sulfur subunit (WP_013416316.1) homologous to succinate:menaquinone oxidoreductase SdhB from *Bacillus licheniformis* (Q65GF5) was identified through comparison with TCDB. Within the retrieved *Rhodococcus* genomes, this subunit was found primarily in clinical genomes (Table 1). In the *R. equi* 103S genome, the gene encoding WP_013416316.1 was located in a gene cluster (NC_014659.1:2876917–2880771) alongside genes encoding a putative succinate dehydrogenase flavoprotein subunit (WP_041674290.1) and two hypothetical proteins (WP_013416318.1, WP_005512616.1) (Fig. 3A). In this gene cluster and its genomic neighborhood, genes encoding for the succinate dehydrogenase transmembrane subunit were not found.

Domain predictions for hypothetical proteins WP_013416318.1 and WP_005512616.1 were carried out using both CD-Search and InterProScan, but no functional domains were identified. InterProScan also performs transmembrane topology prediction through PHOBIUS. Using this tool, cytoplasmic, transmembrane, and non-cytoplasmic regions were predicted in the hypothetical protein WP_013416318.1 (Fig. 3C). BLAST search of the “nr” database of NCBI revealed that the hypothetical protein was homologous to the transmembrane subunit of the succinate dehydrogenase SDH1 found in *M. tuberculosis* H37Rv (e-value=1e^-144^; identity = 70.45%; coverage = 99%). Genomic comparisons demonstrated that *R. equi* 103S putative SDH1 (NC_014659.1:2876917–2880771) and SDH2 (NC_014659.1:3643203–3646595) are syntenic to *M. tuberculosis* H37Rv SDH1 (NC_000962.3:298116–302028) and SDH2 (NC_000962.3:3704102–3707563) loci (Fig. 3A and B).

### Two Ni-Fe Hydrogenase complexes with distinct functions

In the *R. equi* 103S genome, there are two predicted catalytic subunits of Ni-Fe hydrogenases, which were identified as homologs of respiratory complex proteins using TCDB. Previous phylogenetic studies of these enzymes classified the two Ni-Fe hydrogenases found in *R. equi*   103S into two distinct groups, 3b and 1h (Greening et al. 2016). From here on, this nomenclature will be used to refer to these different proteins.

Comparisons with TCDB revealed that the hydrogenase 3b catalytic subunit (WP_005514364.1) is homologous to the membrane-bound F420 non-reducing hydrogenase subunit A (P60227) from the archaeon *Methanothermobacter marburgensis* (Table 1). In the genome of *R. equi* 103S, the ORF encoding the hydrogenase 3b catalytic subunit was found in a gene cluster (NC_014659.1:1580905–1585950) with five other ORFs, which are predicted to encode an oxidoreductase (WP_013415447.1), an FAD/NAD(P)-binding protein (WP_013415448), a cyclic nucleotide-binding domain-containing protein (WP_013415449.1), a protein containing 4Fe-4 S clusters (WP_041674071.1), and a hydrogenase maturation protein HyaD (WP_049799448.1) (Fig. 3D). The ORF encoding the putative oxidoreductase WP_013415447.1 was also identified as a homolog of the subunit G from *M. marburgensis* F420 non-reducing hydrogenase (P60239) via comparisons with TCDB (Table 1). No homolog for the membrane-anchoring subunit D (P60238) was found in the *R. equi* 103 S genome.

In *M. smegmatis* (Berney et al. 2014b) *Pyrococcus* *furiosus* (Ma and Adams 2001), hydrogenase 3b was experimentally characterized as being constituted of four subunits. Sequence alignments through BLAST with the hydrogenase 3b from these organisms demonstrated that *R. equi* 103S putative hydrogenase 3b catalytic subunit (WP_005514364.1), protein containing 4Fe-4 S clusters (WP_041674071.1), putative oxidoreductase (WP_013415447.1), and hydrogenase maturation protein HyaD (WP_013415448.1) are homologous to the HydA, HydB, HydD, and HydG subunits, respectively, from *M. smegmatis* and *P. furiosus* (Fig. 3D, Table S9).

The *R. equi* putative hydrogenase 1 h catalytic subunit was found to be homologous to the HydB catalytic subunit of HydABC from *Wolinella succinogenes* (Table 1), which is also a Ni-Fe hydrogenase but is classified as part of group 1b (Greening et al. 2016). Prediction with CD-search shows that both proteins share the pfam00374 domain. However, homologs for HydA and HydC subunits of *W. succinogenes* were not found in *R. equi* 103S.

Group 1 h hydrogenases have previously been characterized in *M. smegmatis* (Cordero et al. 2019) and *Cupriavidus necator* (Schäfer et al. 2016), where they are predicted to be constituted of a catalytic large subunit (HhyL) and a non-catalytic small subunit (HhyS). A BLAST search was performed using the known HhyS from *M. smegmatis* (WP_011728543.1) (Islam et al. 2019) *C.* *necator* (WP_011153986.1) (Schäfer et al. 2016) as queries. Both of these proteins were found to be homologous to the *R. equi* 103S protein WP_013417287.1, which was annotated as a hydrogenase expression protein HypE (Table S10). Comparison with TCDB indicated that *R. equi* 103 S WP_013417287.1 is homologous to the small subunit of the F420 non-reducing hydrogenase I from *Methanosarcina mazei* (Table 1). The gene encoding WP_013417287.1 was found adjacent to the *hhyL* gene (Fig. 3E). Both genes were located in a gene cluster (NC_014659.1:4430343–4445450) containing 10 other genes predicted to encode proteins related to hydrogenase maturation, as well as 6 genes encoding hypothetical proteins. This cluster is also conserved in *M. smegmatis* MC2 155 and *C. necator* H16 (Fig. 3E). Four of the *R. equi* 103S putative hydrogenase maturation proteins (WP_013417278.1, WP_005517454.1, WP_013417281.1, and WP_005517438.1) were identified as homologous to hydrogenase maturation proteins deposited in TCDB (Table 1).

## Discussion

### Nitrate respiration pathway

Comparison with TCDB revealed 67 proteins in the *R. equi* 103S genome that are possibly related to proton transport across the cytoplasmic membrane or in electron transport to the membrane quinone pool, creating a proton-motive force that drives ATP synthesis. These proteins include the canonical aerobic respiration complexes, such as complexes I, III, and IV (Marreiros et al. 2016). Their wide distribution across *Rhodococcus* genomes indicates the importance of the aerobic respiration pathway in these bacteria. On the other hand, 13 of the putative respiratory chain subunits were found only in specific groups, indicating different metabolic adaptations across the genus. Due to the large difference in the number of clinical and non-clinical genomes available in the RefSeq database, statistical inferences between the distribution of genes and their role in virulence was not possible. For example, the nitrate reductase NarGHI complex was found only in genomes from *R. equi*, *R. rhodochrous*, *R. pyridinivorans*, *R. gordoniae*, and *R. ruber*.

The NarGHI complex is a known *R. equi* 103S virulence factor. The conservation of this enzyme complex in *R. equi* may be attributed to its importance for the virulence of these opportunistic pathogens, as previously observed (Pei et al. 2007). During infection, reactive oxygen species and reactive nitrogen species, such as O_2_^−^ and NO, are produced by activated macrophages to eliminate pathogens through oxidative stress (Brüne et al. 2013; Canton et al. 2021; Okda et al. 2025). In the phagosome, O_2_ is quickly consumed to produce O_2_^-^, resulting in a hypoxic environment (Hampton and Dickerhof 2023). O_2_^−^ and NO can react to form peroxynitrite (ONOO^−^), which can subsequently be converted into inert NO_3_^−^ (Szabó et al. 2007). Utilization of host-derived NO_3_^-^ for anaerobic respiration has been observed as an important factor in *E. coli* (Winter et al. 2013) and *Klebsiella pneumoniae* intestinal infection (Xie et al. 2025), as well as opportunistic infections of the lower respiratory tract by *P. aeruginosa* (Palmer et al. 2007) and *Neisseria mucosa* (Toney et al. 2025). *R. equi* is able to survive the oxidative stress during phagocytosis (Mourenza et al. 2019) and may be able to utilize NO_3_^-^ as the final electron acceptor in the oxygen-limited conditions of the phagosome.

The *R. equi* 103S genome is also predicted to encode a nitric oxide reductase (NOR), which has previously been classified as a member of the quinol-dependent NOR (qNOR) family. The utilization of membrane quinols as electron donors is characteristic of qNORs (Murali et al. 2022, 2024). Other qNORs are predicted to pump protons (H^+^) from the cytoplasm to the extracellular space (Gonska et al. 2018; Gopalasingam et al. 2019). If the *R. equi* putative NOR function is similar to the NarGHI complex as an electron acceptor in the respiration chain, it could contribute to the generation of a proton-motive force. Although some bacteria produce NO through reduction of NO_2_^-^ (Zhu et al. 2025), there is no evidence that *R. equi* possesses the necessary dissimilatory nitrite reductase. Therefore, the NO used must be acquired from the environment, most likely from NO produced by host macrophages. The production of NO by activated macrophages has been shown to be an important factor in restricting *R. equi* intracellular growth (Von Bargen et al. 2011). In this scenario, we hypothesize that the reduction of extracellular NO by qNOR can also assist in resistance to NO-mediated toxicity.

While *R. equi* 103S lacks the ability to produce NO from nitrite, it possesses the nitrite reductase NirBD, which catalyzes the reduction of NO_2_^-^ to NH_4_^+^ (Letek et al. 2010). The reduction of NO_2_^-^ is an important step in nitrate reduction pathways to prevent accumulation and potential toxic side effects (Page et al. 1990; Bueno et al. 2018). Alkalinization of phagolysosomal pH by NH_4_^+^ has been reported as an important virulence factor for intraphagolysosomal survival in other pathogens, such as *Helicobacter pylori* (Schwartz and Allen 2006), *Aspergillus fumigatus* (Xiong et al. 2023), *Coccidioides posadasii* (Mirbod-Donovan et al. 2006), and *Mycobacterium bovis* bacillus Calmette-Guérin (BCG) (Mukai et al. 2008).

### Membrane-bound formate dehydrogenase

In addition to these terminal acceptors, membrane complexes that act as electron donors were conserved across the *Rhodococcus* genus. Among them, the most interesting in the context of anaerobic respiration are the homologs of the α and β subunits from the membrane-bound periplasmic FDHs found in *E. coli*. This FDH plays an important role in proton-motive force generation during NO_3_^-^ respiration. It couples the oxidation of extracellular formate with the pumping of H^+^ through the cytoplasmic membrane. The electrons released by formate oxidation are transferred to the quinone pool in the plasma membrane. These electrons are then used by the nitrate reductase NarGHI complex to reduce NO_3_^-^ (Jones 1980; Unden et al. 2014).

Although no homologs of the membrane anchor subunit FDH-γ were found, the predicted 3D structure indicated an α-helix in the C-terminus of the *R. equi* 103S FDH-β subunit (WP_013415246.1), similar to the one found in the *E. coli* FDH-β subunit, where it is buried in the membrane and interacts with the FDH-γ subunit (Jormakka et al. 2002). Together with the subcellular location prediction by PSORTb, these results suggest that *R. equi* 103S FDH is also a heterotrimeric enzyme bound to the membrane but is possibly anchored by a different protein, such as the predicted NfrD family protein (WP_041673985.1) found in the same gene cluster as the FDH-α subunit (WP_162096942.1) and FDH-β subunit. The predicted interactions between the FDH-β subunit and the NfrD family protein in the 3D structure further support this assumption.

In the results of the phylogenetic analysis, we were not able to arrive at an exact classification for the *R. equi* FDH enzyme in the classification system proposed by Nielsen et al. (2019), since the *R. equi* 103S FDH-α subunit was grouped in the same clade as FDH enzymes from different types, including Type 2 FDHs from *M. gigas*, *Desulfovibrio desulfuricans*, and *Syntrophobacter fumaroxidans*, and Type 4 FDHs from *E. coli*, *Nitratideuslfovibrio vulgaris*, and *Pseudomonas aeruginosa.* All the experimentally characterized FDHs within this clade were predicted to possess a Tat signal peptide by SignalP and DEEPTMHMM. Although SignalP did not identify a Tat signal peptide in *R. equi* 103S FDH-α, its N-terminus has the most important features of this signal peptide: the essential AxA motif and the phenylalanine residue in the SRRxFLK motif (Stanley et al. 2000). These results suggest that the *R. equi* 103S FDH-α subunit is exported from the cytoplasm through the Tat system.

### Succinate dehydrogenases

Among other possible electron-donor complexes, two distinct succinate dehydrogenases were identified, homologous to SDH1 and SDH2 from *M. tuberculosis*. Although SDHs share an evolutionary history, they are classified as Class A-F based on the biochemical properties of their membrane-spanning regions, such as the number of membrane anchor subunits and transmembrane helices (Lancaster 2013). *R. equi* 103S SDH1 and SDH2 were identified as homologs to *M. tuberculosis* SDH1 and SDH2, which are classified as Classes F and A, respectively (Gong et al. 2020; Zhou et al. 2021). Due to the high sequence identity to their *M. tuberculosis* homologs, *R. equi* SDHs could be classified in the same classes.

Both SDH enzymes are anchored to the cytoplasmic membrane, transferring electrons to the membrane quinone pool by oxidizing succinate into fumarate (Hards et al. 2019). In *M. tuberculosis*, the activity of both SDH enzymes seems essential for respiratory chain function (Adolph et al. 2022). Although they may be similar, their roles in bacterial respiration do not appear to be redundant. Previous reports suggest that SDH1 acts primarily during aerobic respiration, whereas SDH2 appears to be more important for survival under oxygen-limited conditions (Baek et al. 2011; Hartman et al. 2014). In *M. smegmatis*, a non-pathogenic species of *Mycobacterium*, SDH2 is the main generator of membrane potential under hypoxia, and its activity cannot be compensated by SDH1 (Pecsi et al. 2014). Under anaerobic conditions, some SDHs are also able to perform the reverse reaction, reducing fumarate to succinate, which acts as the terminal electron acceptor (Maklashina et al. 1998; Spinelli et al. 2021). In *M. tuberculosis*, neither SDH1 nor SDH2 exhibited fumarate reductase activity under hypoxia, indicating a distinct influence on the bacterium’s anaerobic metabolism (Pecsi et al. 2014). The presence of both enzymes in *Rhodococcus* genomes may indicate that they also contribute to survival under conditions with varying oxygen availability.

### Ni-Fe hydrogenases

Hydrogen reduction by Ni-Fe hydrogenases is an important energy source during host colonization in other pathogens, such as *Helicobacter pylori* (Olson and Maier 2002) and *Salmonella enterica* (Maier et al. 2004). These enzymes can be separated into different groups, which reflect differences in their composition and functions (Vignais 2001; Vignais and Billoud 2007; Calusinska et al. 2010). Group 3b includes cytosolic hydrogenases that couple NAD(P)H oxidation with H_2_ production (Greening et al. 2016). In *R. equi* 103S, the gene encoding the catalytic subunit is predicted to be a hydrogenase 3b and is located in a gene cluster along with genes encoding an oxidoreductase, a protein with an FAD/NAD(P)-binding domain, and a protein containing 4Fe-4 S centers, all of which were shown to be homologous to the subunits found in *M. smegmatis* (Berney et al. 2014b). These proteins are also homologous to hydrogenase 3b from the archaeon *P. furiosus* (Ma et al. 2000). In *Proteus mirabilis*, a membrane-bound formate hydrogenlyase couples formate oxidation to H_2_ production, thereby removing H^+^ from the cytoplasm to maintain pH homeostasis under acidic conditions (Lin and Liaw 2020). Although it is known that *R. equi* survival in phagolysosomes depends on VapA, a secreted protein encoded by virulence plasmids, its expression is induced only after the bacterium is phagocytosed by macrophages (Haubenthal et al. 2023). In this scenario, the production of H_2_ by hydrogenase 3b, which uses 2 H^+^ to produce H_2_, could help maintain cytoplasmic pH homeostasis while VapA has yet to be activated.

Group 1h includes membrane-anchored hydrogenases, which scavenge tropospheric H_2_ and use it as an electron donor to sustain respiration under energy-restricted conditions (Greening et al. 2015). Through comparison with other known 1h hydrogenases, it was possible to identify the putative small subunit for this enzyme in *R. equi*. Although membrane fractioning experiments have shown that this type of hydrogenase is membrane-associated, no anchoring membrane subunit has been identified, and its interactions with the membrane remain to be elucidated (Greening et al. 2014). In *Rhodococcus*, this hydrogenase was found in a conserved gene cluster that also included genes predicted to encode hydrogenase maturation proteins present in many other organisms (Constant et al. 2011). This cluster also includes four hypothetical proteins. One of these proteins, containing an NifU domain, was previously found to be essential for the function of Ni-Fe hydrogenase 1 h in *M. smegmatis* (Islam et al. 2019). In the clinical strain *R. equi* ATCC 337707, H_2_ consumption via Ni-Fe hydrogenase 1 h has been shown to correlate with the end of the exponential phase and the beginning of the stationary phase of bacterial growth, suggesting its importance under nutrient-limited conditions (Meredith et al. 2014).

Among the 334 analyzed genomes, loci encoding these hydrogenase complexes showed a high rate of co-occurrence. The co-occurrence of genes encoding 1h and 3b hydrogenases has previously been observed in *M. smegmatis*, along with a hydrogenase 2a (Berney et al. 2014b). *M. smegmatis* hydrogenase 3b oxidation of NAD(P)H produces H_2_, which can be oxidized by hydrogenase 2a, producing 2 H^+^ and transferring electrons to the quinone pool of the plasma membrane (Berney et al. 2014a). As *R. equi* lacks hydrogenase 2a, the production of H_2_ by hydrogenase 3b could be the source of H_2_ for hydrogenase 1h.

**Fig. 4:**
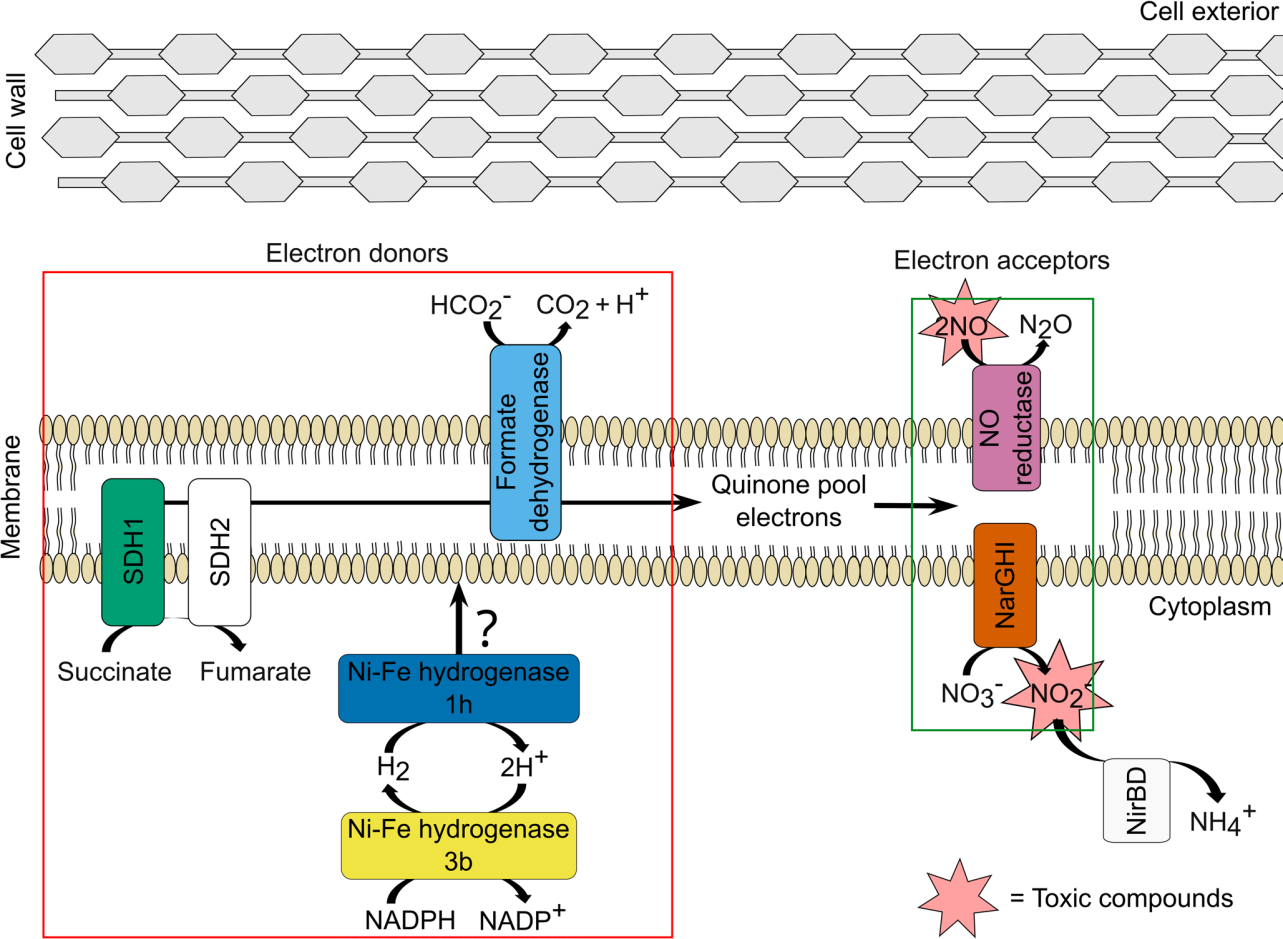
Proposed model of interactions between components of the *Rhodococcus* respiratory chain investigated in this study. *Rhodococcus* were predicted to utilize multiple electron donor and acceptor molecules through a diverse repertoire of enzymes. Formate, succinate, and H_2_ serve as electron donors via a formate dehydrogenase, two succinate dehydrogenases, and two Ni-Fe hydrogenases, respectively. These enzymes transfer electrons into the membrane-associated quinone pool. From the quinone pool, electrons can be transferred to terminal acceptors via nitrate reductase NarGHI and nitric oxide reductase, coupling electron transfer to the generation of proton-motive force. Nitrite, a toxic byproduct of NarGHI-mediated nitrate reduction, is detoxified by NirBD through its conversion to ammonia. Similarly, the reduction of nitric oxide by nitric oxide reductase serves a dual role in energy conservation and in protection against the cytotoxic effects of this reactive molecule

Taken together, the results suggest that *R. equi* possesses a varied set of respiratory chain complexes, including the utilization of NO_3_^-^ as a final electron acceptor, that may reflect an adaptation to mammalian hosts. As seen in other bacterial pathogens, such as *E. coli*, the primary electron donor may be formate, with other supplemental sources such as succinate and H_2_ (Fig. 4). The identification of loci encoding multiple respiratory chain complexes indicates that *R. equi* possesses a highly branched respiratory chain. The ability to utilize different substrates as electron donors and electron acceptors may facilitate ATP synthesis in environments with varying nutrient availability (Srivastav et al. 2025). Functional contributions of individual complexes to *R. equi* metabolism must still be experimentally validated, as previously demonstrated for NarGHI.

## Supplementary Information

Below is the link to the electronic supplementary material.

Supplementary Material 1

Supplementary Material 2

Supplementary Material 3

## Data Availability

All genomic data used in this study was queried from NCBI’s RefSeq database. The list of used genomes can be consulted on Table S1. Relevant data generated from our analysis, which supports the findings of this study, are available in the supplementary material. Additional data and scripts related to this paper are available from the corresponding author on reasonable request.
