# Supplementary material for: Adaptations in electron transport chain complexes of clinical Rhodococcus equi revealed through comparative genomics
Source: World J Microbiol Biotechnol. 2026 Jul 4;42(7):390. doi: 10.1007/s11274-026-05110-w (PMC13332983; doi:10.1007/s11274-026-05110-w)
Supplement: Supplementary file 1 — Supplementary Material 1 [file 11274_2026_5110_MOESM1_ESM.pdf]

Table S3

Genomes from RefSeq database used to perform the comparison and synteny analysis with the pathogenic reference strain *R. equi* 103S.

Organism	RefSeq accession ID	Reference
Rhodococcus equi 103S	GCF_000196695.1	Letek et al. (2010)
Mycobacterium smegmatis MC2 155	GCF_000767605.1	Mohan et al. (2015)
Pyrococcus furiosus DSM 3638	GCF_008245085.1	-
Cupriavidus necator H16	GCF_000009285.1	Pohlmann et al. (2006)
Mycobacterium tuberculosis H37Rv	GCF_000195955.2	Camus et al. (2002)
Rhodococcus rhodochrous EP4	GCF_003004765.2	-

Table S7

PSORTb and DeepTMHMM results for putative FDH subunits from *R. equi* 103S.

Subunit	RefeSeq Protein Accession	PSORTb		DeepTMHMM	
		Subcellular location	Internal helices	Protein types	Transmembrane regions
FDH-α	WP_162096942.1	Cytoplasmic	0	Globular with Signal peptide	0
FDH-β	WP_013415246.1	Cytoplasmic Membrane	0	Alpha helical transmembrane	1
NrfD family protein	WP_041673985.1	Cytoplasmic Membrane	6	Alpha helical transmembrane	8

Table S8

CD-Search results identify the subunits from the twin-arginine translocase (Tat) system found in *R. equi* 103S.

Tat subunit	*R. equi* 103S RefSeq accession ID	CDD specific hit	E-value	Conserved domain length
TatA	WP_005516546.1	PRK00575	8.18e-29	92
	WP_005513218.1	PRK00575	3.20e-36	92
TatB	WP_013415394.1	TIGR01410	2.34e-21	80
TatC	WP_013416026.1	COG0805	3.53e-86	249

Table S9

BLAST results between *R. equi* 103S putative Ni-Fe hydrogenase 3b subunits and experimentally characterized Ni-Fe hydrogenase 3b subunits from *M. smegmatis* MC2 155 and *P. furiosus* DSM 3638.

Ni-Fe hydrogenase 3b subunit	*R. equi* 103S RefSeq accession	*R. equi* 103S protein length (amino acids)	Organism used in comparison	Homologous protein RefSeq accession	Homologous protein length (amino acids)	Identity (%)	*R. equi* 103S coverage (%)	E-value
HydA	WP_005514364.1	439	*M smegmatis*	WP_003895383.1	430	64.5	98	0
			P. furiosus	WP_011012029.1	428	31.0	95	$1.77e^{-74}$
HydB	WP_041674071.1	383	*M smegmatis*	WP_042510581.1	367	55.9	89	$3.64e^{-122}$
			P. furiosus	WP_011012026.1	367	29.9	77	$2.67e^{-34}$
HydD	WP_013415447.1	263	*M smegmatis*	WP_003895384.1	252	67.4	91	$2.55e^{-120}$
			P. furiosus	WP_011012028.1	261	33.9	90	$7.01e^{-48}$
HydG	WP_013415448.1	277	*M smegmatis*	WP_174519602.1	279	50.4	86	$6.73e^{-69}$
			P. furiosus	WP_011012027.1	292	32.6	96	$1.27e^{-48}$

Table S10

BLAST results for search of HhyS subunit in *R. equi* 103S using HhyS sequences from *M. smegmatis* and *C. necator*.

Organism	Query sequence RefSeq accession	Query sequence length (amino acids)	*R. equi* 103S RefSeq accession	*R. equi* 103S protein length (amino acids)	Identity (%)	Query coverage (%)	E-value
M. smegmatis	WP_011728543.1	351	WP_013417285.1	361	81.4	99	0
C. necator	WP_011153986.1	351			67.9	93	$3e^{-177}$

Supplementary References

Camus, J.-C., Pryor, M.J., Médigue, C., Cole, S.T., 2002. Re-annotation of the genome sequence of *Mycobacterium tuberculosis* H37Rv. Microbiol. Read. Engl. 148, 2967–2973. https://doi.org/10.1099/00221287-148-10-2967

Letek, M., González, P., MacArthur, I., Rodríguez, H., Freeman, T.C., Valero-Rello, A., Blanco, M., Buckley, T., Cherevach, I., Fahey, R., et al., 2010. The genome of a pathogenic *Rhodococcus*: cooptive virulence underpinned by key gene acquisitions. PLoS Genet. 6, e1001145. https://doi.org/10.1371/journal.pgen.1001145

Mohan, A., Padiadpu, J., Baloni, P., Chandra, N., 2015. Complete genome sequences of a *Mycobacterium smegmatis* laboratory strain (MC2 155) and isoniazid-resistant (4XR1/R2) Mutant Strains. Genome Announc. 3, e01520-14. https://doi.org/10.1128/genomeA.01520-14

Pohlmann, A., Fricke, W.F., Reinecke, F., Kusian, B., Liesegang, H., Cramm, R., Eitinger, T., Ewering, C., Pötter, M., Schwartz, E., et al., 2006. Genome sequence of the bioplastic-producing “Knallgas” bacterium Ralstonia eutropha H16. Nat. Biotechnol. 24, 1257–1262. https://doi.org/10.1038/nbt1244

van den Heuvel, R.H.H., Curti, B., Vanoni, M.A., Mattevi, A., 2004. Glutamate synthase: a fascinating pathway from L-glutamine to L-glutamate. Cell. Mol. Life Sci. CMLS 61, 669–681. https://doi.org/10.1007/s00018-003-3316-0
