# Supplementary material for: Adaptations in electron transport chain complexes of clinical Rhodococcus equi revealed through comparative genomics
Source: World J Microbiol Biotechnol. 2026 Jul 4;42(7):390. doi: 10.1007/s11274-026-05110-w (PMC13332983; doi:10.1007/s11274-026-05110-w)
Supplement: Supplementary file 3 — Supplementary Material 3 [file 11274_2026_5110_MOESM3_ESM.pdf]

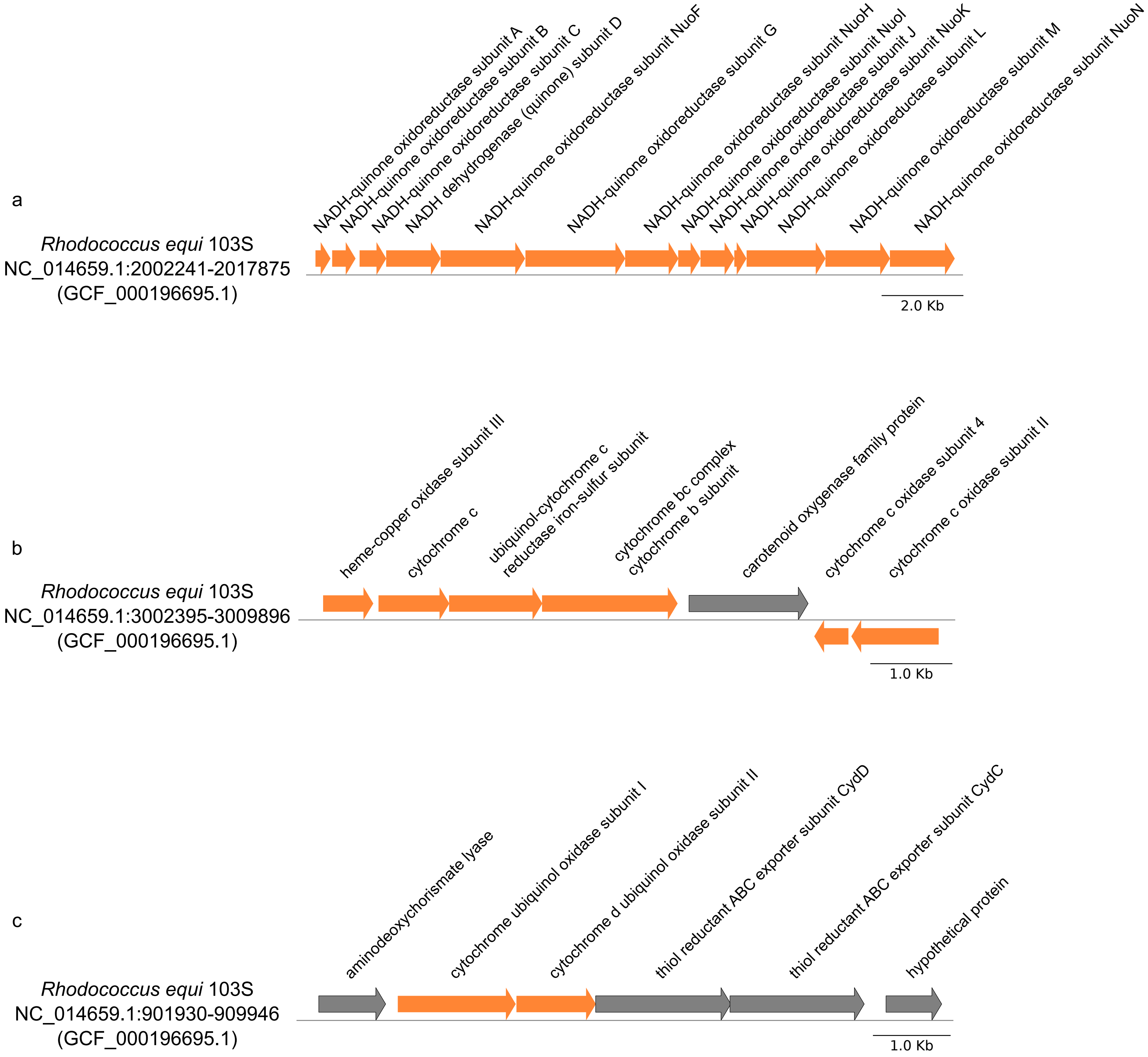

Fig. S1 Gene clusters in *Rhodococcus equi* 103S encoding for aerobic respiration electron transport complexes. **a:** NADH-quinone oxidoreductase. **b:** cytochrome c oxidase. **c:** cytochrome bd oxidase. Each arrow represents a gene. Orange arrows are genes predicted to encode subunits for their respective respiratory complexes. The functional annotation for each gene was retrieved from the RefSeq database

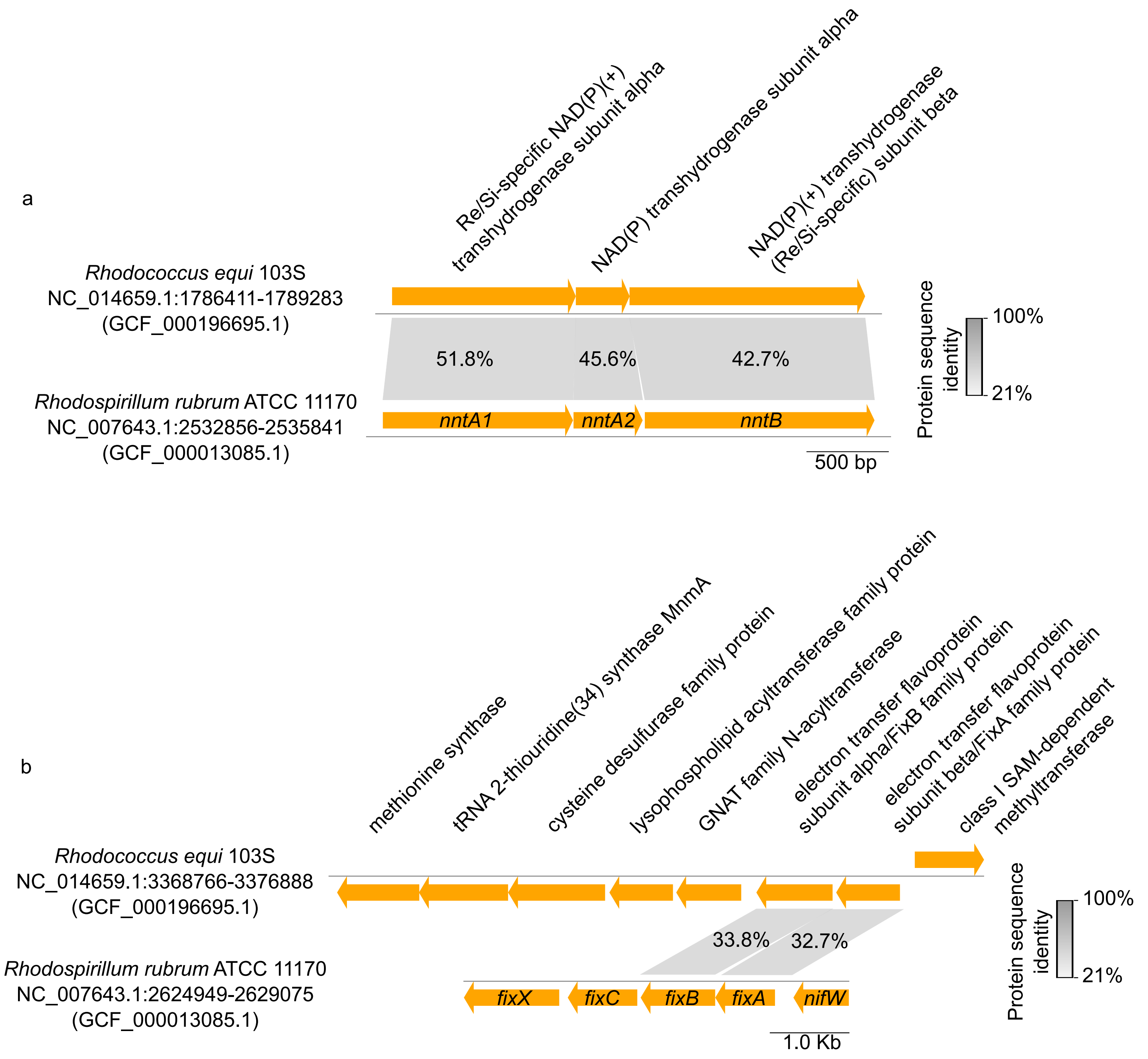

Fig. S2 Genomic comparison between *Rhodococcus equi* 103S and *Rhodospirillum rubrum* ATCC 11170. **a:** NAD(P)(+) transhydrogenase gene cluster. **b:** FixABCX complex gene cluster. Each arrow represents a gene. Grey trapeziums drawn between sequences indicate the reciprocal best hits relationship between encoded proteins. The grey scale color of the trapeziums represents the sequence identity value between protein amino acid sequences. The functional annotation for each gene was retrieved from the RefSeq database.

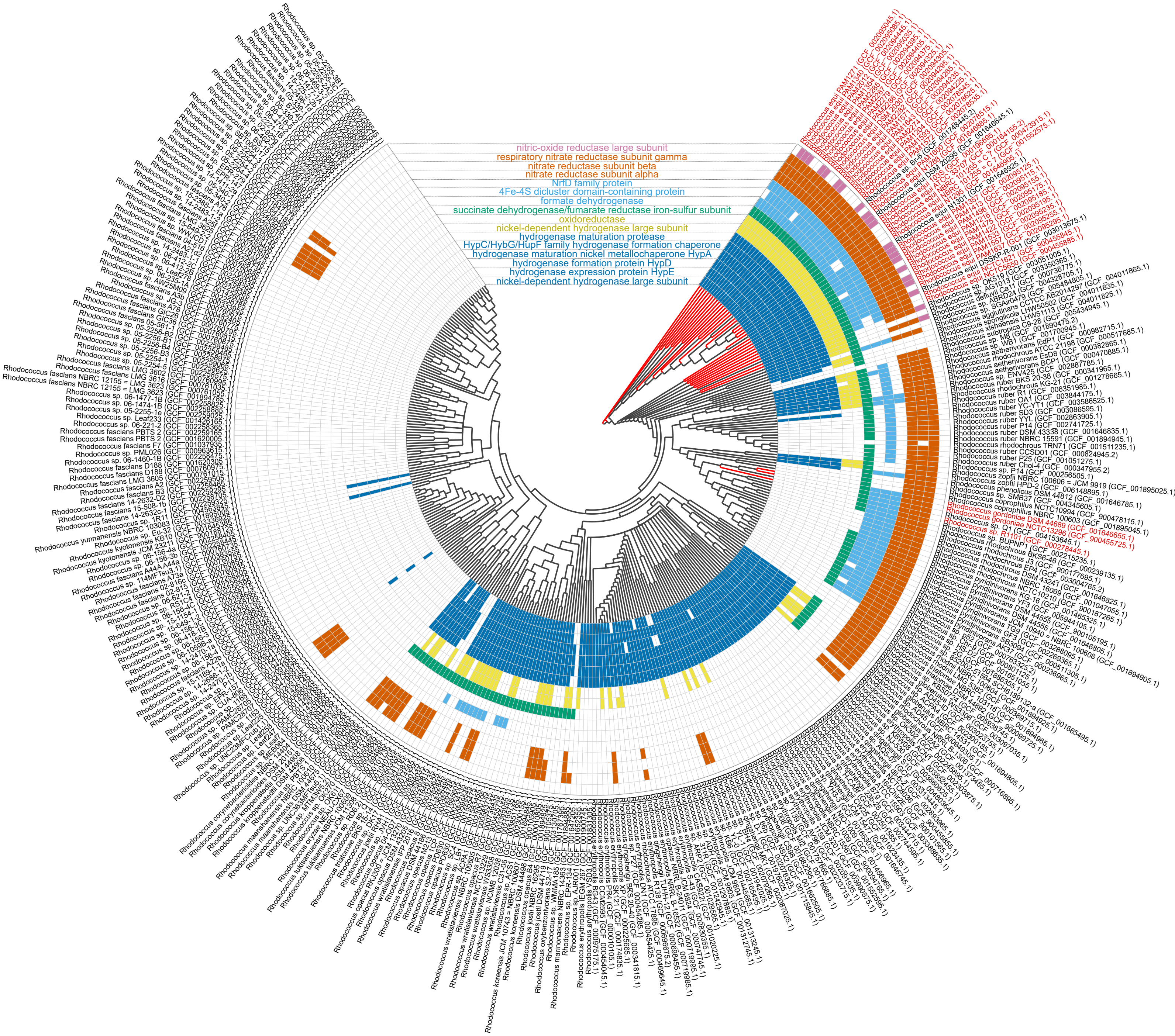

Fig. S3 Distribution along the *Rhodococcus* phylogenetic tree of proteins homologous to TCDB respiratory complexes found in less than 50% of non-clinical genomes. Othofinder species phylogenetic tree inferred for 334 *Rhodococcus* genomes. Label colors indicate the isolation source origin of the genome. Red: clinical origin; Black: non-clinical origin. Each row in the heatmap represents a different protein and their colors represent the protein complexes they are predicted to be a part of. Pink: nitric-oxide reductase; Orange: nitrate reductase NarGHI; Light blue: formate dehydrogenase; Green: Succinate dehydrogenase SDH1; Yellow: Ni-Fe hydrogenase 3b; Dark blue: Ni-Fe hydrogenase 1h.

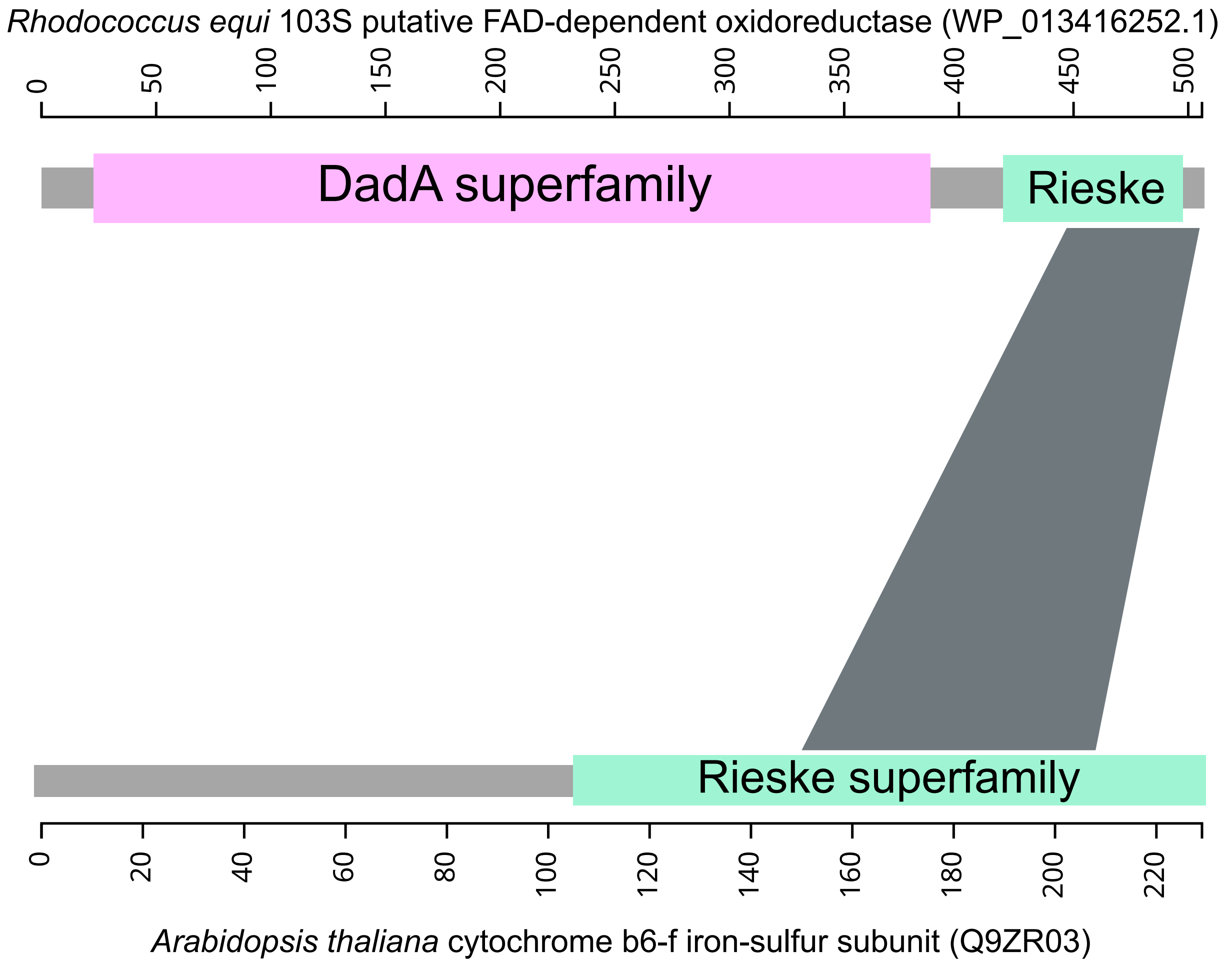

Fig. S4 Alignment between putative FAD-dependent oxidoreductase from *Rhodococcus equi* 103S (top track) and cytochrome b6-f iron-sulfur subunit from *Arabidopsis thaliana* (bottom track). Colored rectangles represent domains predicted using NCBI's CD-Search, Arch. ID 11429760. Accession identifiers in the Conserved Domains Database for the DadA superfamily and Rieske superfamily domains are COG0665 and cd03477, respectively. The grey trapezium drawn between sequences indicates the aligned portions of the sequences. Alignment corresponds mainly to part of the Rieske domain, characterized by the binding of a 2Fe-2S cluster

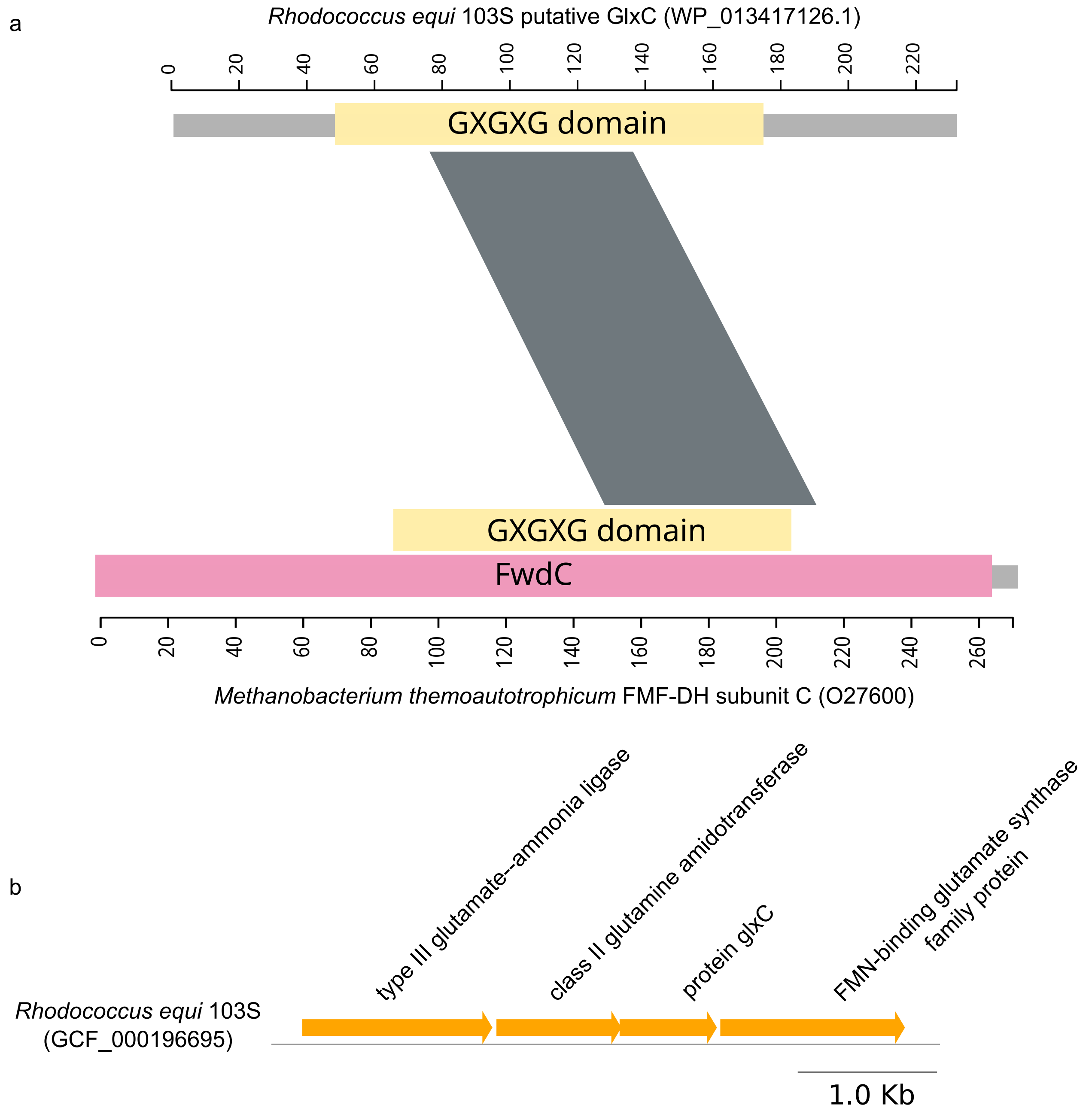

Fig. S5 Analysis of putative glxC protein from *Rhodococcus equi* 103S as a respiratory chain subunit. **a:** Alignment between putative GlxC (top track) and formyl-methanofuran dehydrogenase subunit C from *Methanobacterium themoautotrophicum* (bottom track). Colored rectangles represent domains predicted using NCBI's CD-Search. The grey trapezium drawn between sequences indicates the aligned portions of the sequences. Alignment corresponds mainly to part of the domain of unknown function GXGXG, also found in the large subunit of glutamate synthase, a flavoprotein that catalyzes the synthesis of L-glutamate from L-glutamine and 2-oxoglutarate (van den Heuvel et al., 2004). **b:** Genomic context for the gene encoding the putative GlxC in *Rhodococcus equi* 103S genome. Includes three other genes predicted to encode proteins related to glutamate and glutamine synthesis. Each arrow represents a gene. The displayed gene functional annotations were retrieved from the RefSeq database

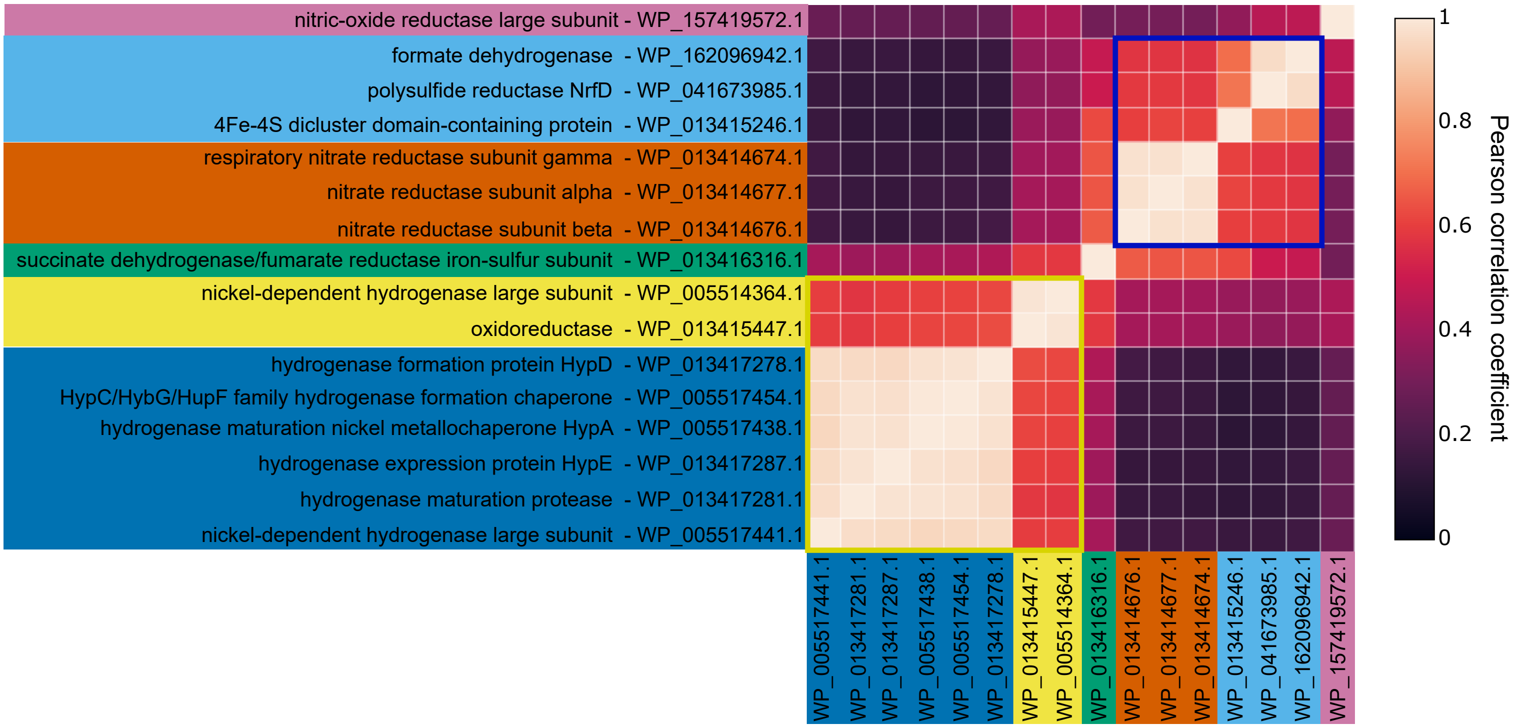

Fig. S6 Co-occurrence patterns of predicted respiratory chain genes across 334 *Rhodococcus* genomes, presented as a heatmap of pairwise Pearson correlation coefficient. Higher values represent a higher rate of co-occurrence between genes. Values are symmetrically distributed along the diagonal. Labels present the RefSeq protein identifiers found in *R. equi* 103S and its predicted functional annotations. Label colors indicate the systems each protein is predicted to be associated with. Pink: nitric-oxide reductase; Orange: nitrate reductase NarGHI; Light blue: formate dehydrogenase; Green: Succinate dehydrogenase SDH1; Yellow: Ni-Fe hydrogenase 3b; Dark blue: Ni-Fe hydrogenase 1h

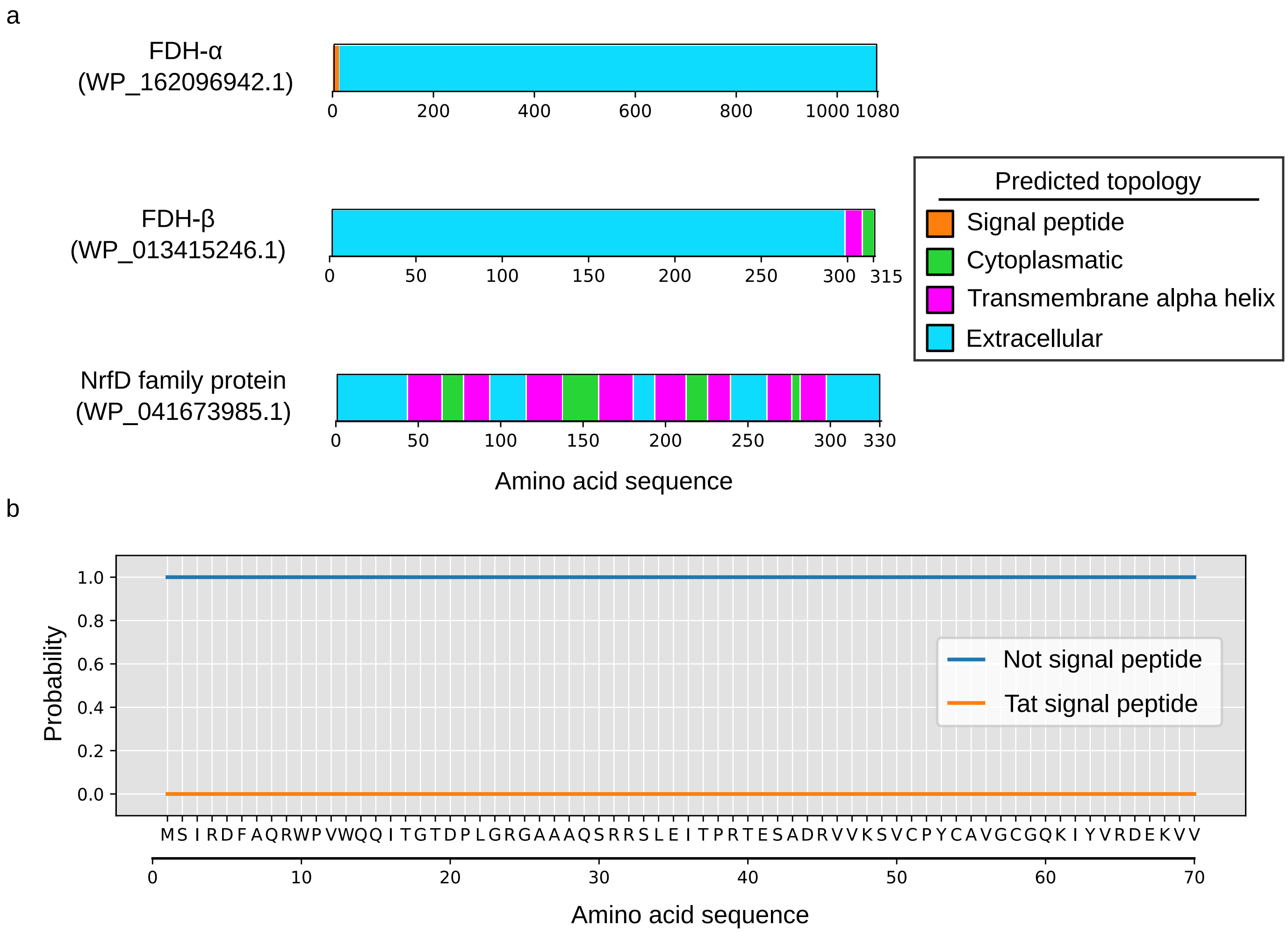

Fig. S7 Signal peptide and topology predictions for *Rhodococcus equi* 103S FDH putative subunits. **a:** Protein topology predicted by DeepTMHMM for putative FDH-α, FDH-β and NrfD family protein. Colors represent the predicted topology in a given region. Orange: signal peptide; Green: cytoplasmic; Magenta: transmembrane alpha helix; Cyan: extracellular. **b:** SignalP probability scores for presence of signal peptide within the first 70 amino acids of the putative FDH-α. Blue line: Not signal peptide; Orange line: Tat signal peptide

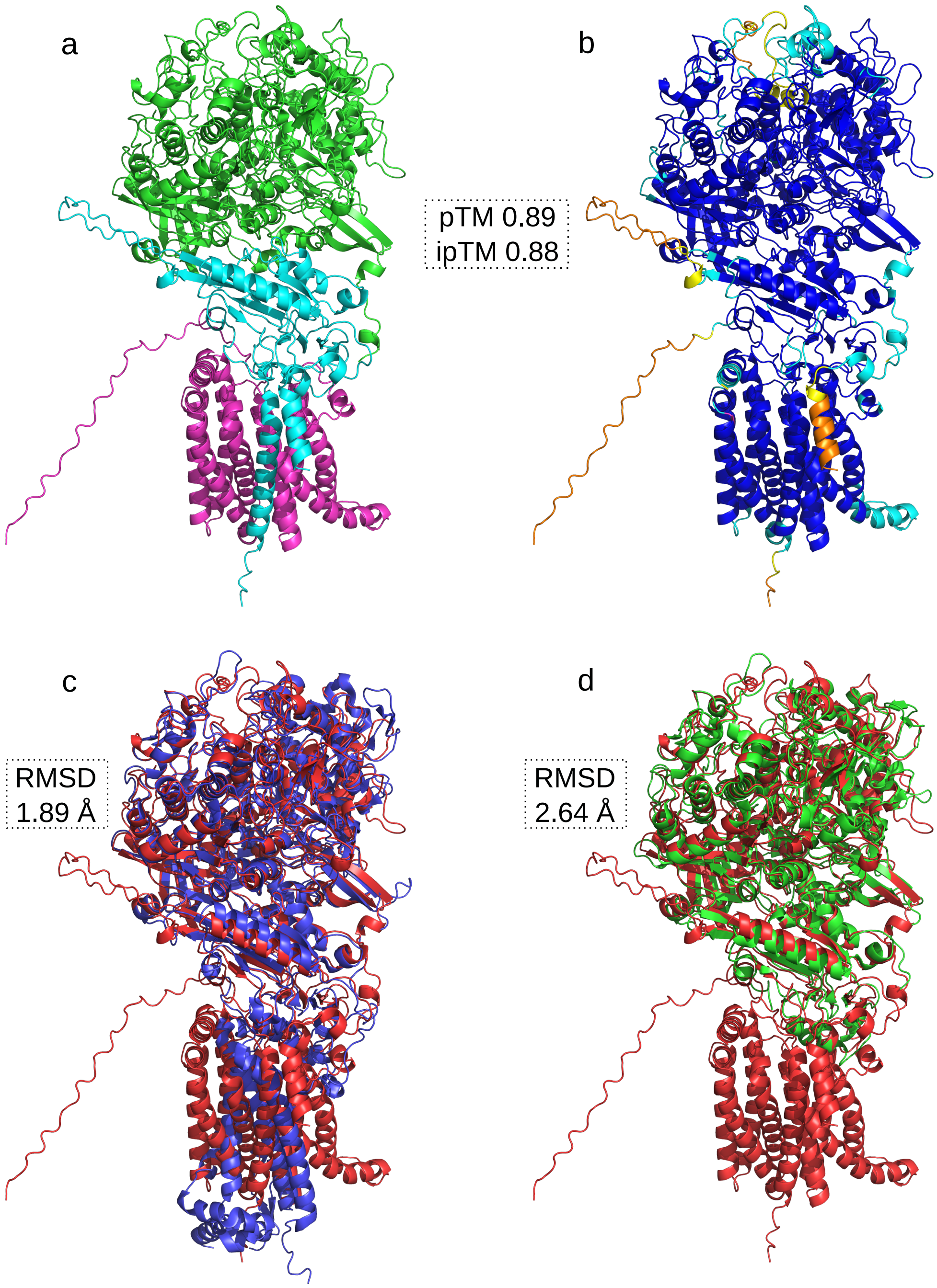

Fig. S8 *Rhodococcus equi* 103S formate dehydrogenase 3D structure as predicted by AlphaFold3. **a:** *R. equi* 103S FDH Structure with colors representing the different subunits. Green: FDH-α; Cyan: FDH-β; Magenta: NrfD family protein. **b:** R. equi 103S FDH structure with colors representing the value for the local confidence metric plDDT. Blue: Very high confidence (plDDT > 90); Cyan: High confidence (90 > plDDT >70); Yellow: Low confidence (70 > plDDT > 50); Orange: Very low confidence (50 > plDDT). **c:** Structural alignment between *R. equi* 103S FDH (red) and *Escherichia coli* FDH-N (blue) (PDB 1KQF). **d:** Structural alignment between *R. equi* 103S FDH (red) and *Megalodesulfovibrio gigas* FDH (green) (PDB 1H0H)
